# Sham Electroacupuncture Methods in Randomized Controlled Trials

**DOI:** 10.1038/srep40837

**Published:** 2017-01-20

**Authors:** Zi-xian Chen, Yan Li, Xiao-guang Zhang, Shuang Chen, Wen-ting Yang, Xia-wei Zheng, Guo-qing Zheng

**Affiliations:** 1 Department of Neurology, the Second Affiliated Hospital and Yuying Children’s Hospital of Wenzhou Medical University, Wenzhou 325027, China

## Abstract

Sham electroacupuncture (EA) control is commonly used to evaluate the specific effects of EA in randomized-controlled trials (RCTs). However, establishing an inert and concealable sham EA control remains methodologically challenging. Here, we aimed to systematically investigate the sham EA methods. Eight electronic databases were searched from their inception to April 2015. Ten out of the 17 sham EA methods were identified from 94 RCTs involving 6134 participants according to three aspects: needle location, depth of needle insertion and electrical stimulation. The top three most frequently used types were sham EA type A, type L and type O ordinally. Only 24 out of the 94 trials reported credibility tests in six types of sham EA methods and the results were mainly as follows: sham EA type A (10/24), type B (5/24) and type Q (5/24). Compared with sham EA controls, EA therapy in 56.2% trials reported the specific effects, of which the highest positive rate was observed in type N (3/4), type F (5/7), type D (4/6) and type M (2/3). In conclusion, several sham EA types were identified as a promising candidate for further application in RCTs. Nonetheless, more evidence for inert and concealable sham EA control methods is needed.

A randomized controlled trial (RCT) has been the cornerstone of medical clinical research since the first RCT paper entitled “Streptomycin treatment of pulmonary tuberculosis: a Medical Research Council investigation” was published in 1948^1^. By the late 20th century, RCT was recognized as the gold standard for a clinical trial^2^. To improve the quality of clinical research, the methodology has been refined to avoid any bias over the past several decades. The most important design techniques for avoiding bias in clinical trials are randomization and blinding. Blinding is intended to limit the occurrence of conscious and unconscious bias in clinical trials (performance bias) conduction and interpretation of outcomes (ascertainment bias)^3^. Blinding is crucial for treatment evaluation because lack of blinding can bias the reliable assessment of treatment effects. For RCTs, placebo is a standard control method to blind the participants and health care providers. The purpose of placebo group is to account for the placebo effect, i.e., effects from treatment that do not depend on the treatment itself. However, blinding is difficult to ensure in non-pharmacological treatment trials because fabrication of placebo such as placebo/sham acupuncture controls requires the placebo to be both inert and indistinguishable, which is relatively difficult^4^.

RCTs for acupuncture appeared in 1970s^5^. Since then, a number of RCTs on acupuncture have been published^6^. The “sham” acupuncture is identified as the procedure controlling for the acupuncture treatment components with the aim to blind the participants and control for non-specific placebo effects^7^. Since participants are to a large extent ignorant of the components of acupuncture such as needle location, depth of needle insertion, needle stimulation and patient/practitioner interactions, sham acupuncture can be considered to be therapeutically inactive. However, it is difficult to design a standard method for sham acupuncture avoiding all therapeutically active components. Thus, the methodological difficulties in designing appropriate sham acupuncture controls for RCTs remained challenging^8^,^9^,^10^.

Electroacupuncture (EA) is an extension technique based on traditional acupuncture combined with modern electrotherapy^11^,^12^. Owing to its accurate, reproducible and standardized intensity and duration of stimulation with simple, verifiable electrical parameters, EA has been widely used in clinical studies and basic research into underlying mechanisms of acupuncture treatment^13^,^14^. Currently, EA is being used extensively in China and elsewhere around the world. However, no systematic analyses have yet been published to describe the sham EA procedures. Thus, the objective of this study is to investigate the sham EA methods utilized in EA RCTs.

## Methods

### Search strategy

Eight electronic databases, including Cochrane Controlled Trials Register, PubMed, EMBASE, AMED, China National Knowledge Infrastructure (CNKI), VIP Journals Database, Wanfang database, and Chinese Biomedical Database (CBM) were searched from their inceptions to April 2015. The search terms were confined to “Electroacupuncture” AND “sham acupuncture OR placebo acupuncture” AND “randomized controlled trial (RCT)”. All searches were limited to studies on human.

### Eligibility Criteria

RCTs concerning the effects of EA on any kind of diseases with at least one control group receiving sham EA were included, regardless of publication status and languages. Quasi-RCTs and non-RCTs were excluded.

The studies were eligible if EA therapy alone or adjunct therapy were given in treatment group and secondly, if sham EA or any type of faked manipulation mimicking real EA in aspects of acupoint, penetration and electro-stimulation were given in control group. There were no restrictions on needle parameters or intensity, frequency and mode of stimulation. Studies that compared EA with transcutaneous nerve electrical stimulation (TNES), another acupuncture plus sham EA or placebo medications were excluded. If three or more groups were designed in one study, only real EA versus sham EA groups were included.

### Study selection and data extraction

Two authors (ZXC, YL) reviewed the titles and abstracts of the potential references independently. All the potentially relevant studies were marked and their full articles were retrieved. Further examinations were carried out to make a final selection decision. The same two authors performed the data extraction independently for the predefined items: author, year, country, EA indications, sample size, the characteristics of interventions, outcome measures, results and dropouts. The disagreements were resolved through consulting a third part (GQZ).

### Risk of bias assessment

Two authors (ZXC and YL) performed the methodological quality assessment of each included trial independently based on the Cochrane Collaboration’s tool for assessing risk of bias^14^. The criteria consisted of the following: adequate sequence generation, allocation concealment, blinding of participants, blinding of personnel, blinding of outcome assessor, free of incomplete outcome data, free of selective reporting and free of other bias.

### Description of sham EA methods

The sham EA methods used in each control group were examined and the details were extracted according to three respects: needle location, depth of needle insertion and electrical stimulation. Partially based on the previous sham acupuncture type I~V classification published by Dincer *et al*.^8^ we summarized seventeen kinds of sham EA methods: (1) Sham EA on therapeutic acupoints plus no skin penetration plus no electrical stimulation (Sham EA type A); (2) Sham EA on therapeutic acupoints plus no skin penetration plus electrical stimulation (Sham EA type B); (3) Sham EA on therapeutic acupoints plus the same depth plus no electrical stimulation (Sham EA type C); (4) Sham EA on therapeutic acupoints plus superficial insertion plus no electrical stimulation (Sham EA type D); (5) Sham EA on therapeutic acupoints plus superficial insertion plus electrical stimulation (Sham EA type E); (6) Sham EA on nonspecific acupuncture points plus the same depth plus electrical stimulation (Sham EA type F); (7) Sham EA on nonspecific acupuncture points plus the same depth plus no electrical stimulation (Sham EA type G); (8) Sham EA on nonspecific acupuncture points plus superficial insertion plus electrical stimulation (Sham EA type H); (9) Sham EA on nonspecific acupuncture points plus superficial insertion plus no electrical stimulation (Sham EA type I); (10) Sham EA on nonspecific acupuncture points plus no skin penetration plus electrical stimulation (Sham EA type J); (11) Sham EA on nonspecific acupuncture points plus no skin penetration plus no electrical stimulation (Sham EA type K); (12) Sham EA on non-acupuncture points plus the same depth plus electrical stimulation (Sham EA type L); (13) Sham EA on non-acupuncture points plus the same depth plus no electrical stimulation (Sham EA type M); (14) Sham EA on non-acupuncture points plus superficial insertion plus electrical stimulation (Sham EA type N); (15) Sham EA on non-acupuncture points plus superficial insertion plus no electrical stimulation (Sham EA type O); (16) Sham EA on non-acupuncture points plus no skin penetration plus electrical stimulation (Sham EA type P); (17) Sham EA on non-acupuncture points plus no skin penetration plus no electrical stimulation (Sham EA type Q).

### Assessment of the effectiveness

Considering wildly varying outcome measures across different disease conditions, treatment efficacy was evaluated for each study according to the modified method based on a previous publication^10^. The results of each trial were presented by using the following primary outcome measures: “T > C” meaning that real EA treatment group was significantly superior to sham EA control group; “ND” meaning no difference between EA and sham EA groups; “T < C” meaning that real EA group was significantly inferior to sham EA group. If the efficacy of a trial was reported as “T > C” or “T < C” without between-groups comparison having been conducted, we collected the original data by reviewing the articles or contacting the corresponding author. If the original data were available, an effect-size analysis was conducted to reconfirm the between-groups difference. If the original data were not available, the efficacy results were presented as “T > C?” or “T < C?”.

### The credibility of blinding

The credibility test was formally performed in validation studies to assess the blinding effect of sham acupuncture based on credibility questionnaire and statistical analysis^15^,^16^,^17^. The information on the credibility test was extracted to explore the relationship between the credibility of blinding and the type of sham EA method.

## Results

### Study selection

**Figure 1:**
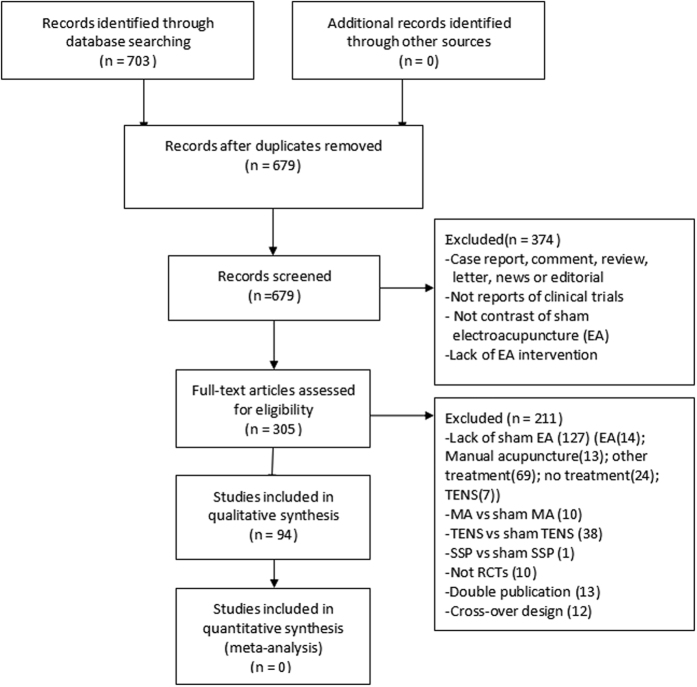
Flow diagram.

A total of 679 potentially relevant articles were identified. By reviewing titles and abstracts, 374 papers were excluded for at least one of following reasons: (1) not clinical trials; (2) case report, comment, review, letter, news or editorial; (3) not in contrast with sham EA; (4) lack of EA intervention. After examining the full content of the remaining 305 articles, we removed 211 records, of which 127 articles were due to lack of sham EA controls, including electro-acupuncture (14 studies), manual acupuncture (13 studies), TNES (7 studies), other treatment (69 studies) or no treatment (24 studies); 49 articles removed for lack of real EA groups, with their target intervention designed as manual acupuncture (10 studies), TNES (38 studies) or periosteal stimulation therapy (PST) (1 study); 10 articles were not RCTs; 13 articles were double publications; 12 articles were cross-over design. Ultimately, 94 studies^18^,^19^,^20^,^21^,^22^,^23^,^24^,^25^,^26^,^27^,^28^,^29^,^30^,^31^,^32^,^33^,^34^,^35^,^36^,^37^,^38^,^39^,^40^,^41^,^42^,^43^,^44^,^45^,^46^,^47^,^48^,^49^,^50^,^51^,^52^,^53^,^54^,^55^,^56^,^57^,^58^,^59^,^60^,^61^,^62^,^63^,^64^,^65^,^66^,^67^,^68^,^69^,^70^,^71^,^72^,^73^,^74^,^75^,^76^,^77^,^78^,^79^,^80^,^81^,^82^,^83^,^84^,^85^,^86^,^87^,^88^,^89^,^90^,^91^,^92^,^93^,^94^,^95^,^96^,^97^,^98^,^99^,^100^,^101^,^102^,^103^,^104^,^105^,^106^,^107^,^108^,^109^,^110^,^111^ involving 6134 participants were selected (Fig. 1).

### Characteristics of included studies

**Figure 2:**
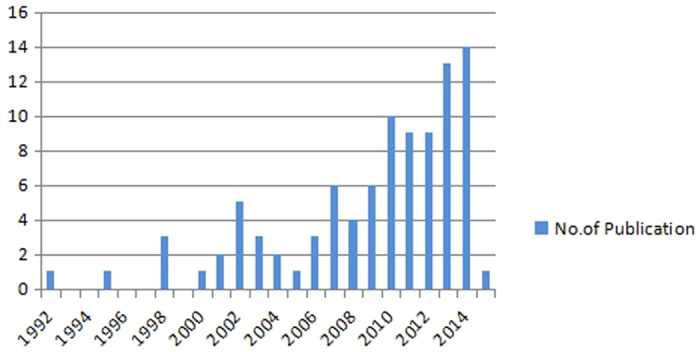
The time distribution of the included articles.

The 94 included articles were published from 1992 to 2015. Among them, 5 studies^29^,^54^,^90^,^95^,^107^ were published between 1992 and 1999; 33 studies^22^,^24^,^25^,^27^,^30^,^31^,^38^,^45^,^47^,^52^,^53^,^56^,^57^,^58^,^67^,^68^,^71^,^72^,^73^,^74^,^77^,^78^,^81^,^83^,^84^,^92^,^93^,^97^,^104^,^105^,^106^,^110^,^111^ were published between 2000 and 2010; the remaining 56 studies^18^,^19^,^20^,^21^,^23^,^26^,^28^,^32^,^33^,^34^,^35^,^36^,^37^,^39^,^40^,^41^,^42^,^43^,^44^,^46^,^48^,^49^,^50^,^51^,^55^,^59^,^60^,^61^,^62^,^63^,^64^,^65^,^66^,^69^,^70^,^75^,^76^,^79^,^80^,^82^,^85^,^86^,^87^,^88^,^89^,^91^,^94^,^96^,^98^,^99^,^100^,^101^,^102^,^103^,^108^,^109^ were reported from 2010 to 2015 (Fig. 2). Indications for EA included pain (32 studies)^18^,^19^,^21^,^24^,^26^,^27^,^31^,^33^,^36^,^37^,^40^,^42^,^45^,^47^,^51^,^52^,^56^,^60^,^66^,^67^,^72^,^76^,^82^,^84^,^88^,^90^,^92^,^96^,^97^,^103^,^110^,^111^, anesthesia (8 studies)^46^,^50^,^73^,^74^,^86^,^89^,^94^,^102^, stroke (7 studies)^25^,^28^,^29^,^34^,^62^,^105^,^106^, depression (6 studies)^23^,^53^,^59^,^65^,^68^,^80^, obesity (4 studies)^32^,^49^,^54^,^70^, primary dysmenorrheal/menstrual pain (4 studies)^61^,^98^,^99^,^101^, substance abuse (heroin or smoking) (3 studies)^64^,^95^,^107^, osteoarthritis (2 studies)^22^,^104^, migraine (2 studies)^39^,^78^, nausea and vomiting (2 studies)^38^,^57^, postoperative ileus (2 studies)^35^,^91^, insomnia (2 studies)^63^,^81^, benign prostate hyperplasia (2 studies)^79^,^87^, diabetic mellitus related diseases (2 studies)^83^,^109^, carpal tunnel syndrome (1 study)^100^, rheumatoid arthritis (1 study)^93^, whiplash-associated disorders (1 study)^69^, constipation (1 study)^48^, multiple sclerosis (1 study)^41^, tinnitus (1 study)^20^, auditory hallucination (1 study)^30^, attention deficit hyperactivity disorder (1 study)^44^, polycystic ovary syndrome (1 study)^55^, hot flushes (1 study)^58^, postpartum insufficient lactation (1 study)^71^, cardiac ischemia-reperfusion injury (1study)^75^, stress-related symptoms (1 study)^108^. The rest three studies^43^,^77^,^85^ reported the effects of EA on healthy subjects.

EA treatment alone was adopted in 55 trials^19^,^20^,^21^,^22^,^23^,^24^,^25^,^26^,^28^,^29^,^30^,^31^,^34^,^35^,^36^,^37^,^38^,^39^,^42^,^43^,^53^,^55^,^58^,^60^,^61^,^66^,^68^,^69^,^71^,^72^,^75^,^77^,^78^,^79^,^80^,^83^,^84^,^85^,^86^,^87^,^88^,^89^,^90^,^91^,^95^,^98^,^99^,^100^,^101^,^103^,^104^,^107^,^108^,^110^,^111^, while the interventions of the remaining 39 trials were a combination of EA and western conventional medicine (WCM). Four trials^24^,^47^,^54^,^100^ were designed as two groups of EA, and seven trials^38^,^43^,^61^,^63^,^98^,^99^,^101^ were conducted with two groups of sham EA. Compared with sham EA group, real EA group in 83 studies selected the same number of acupoints; nine studies^25^,^46^,^66^,^89^,^90^,^105^,^106^,^108^,^111^ used more number of acupoints; one study^20^ used less number of acupoints; one study^52^ did not report the number of acupoints. Eight studies^26^,^49^,^54^,^76^,^95^,^96^,^97^,^110^ identified acupoints by using a point detector. The “*deqi*” sensation was required in 65 real EA groups^18^,^19^,^20^,^21^,^22^,^23^,^24^,^25^,^26^,^29^,^30^,^31^,^33^,^34^,^35^,^36^,^39^,^40^,^41^,^43^,^44^,^47^,^48^,^50^,^51^,^56^,^57^,^58^,^59^,^60^,^61^,^62^,^63^,^64^,^66^,^67^,^69^,^71^,^75^,^77^,^78^,^79^,^80^,^81^,^82^,^83^,^85^,^86^,^87^,^90^,^91^,^93^,^98^,^99^,^100^,^101^,^102^,^104^,^105^,^106^,^109^,^111^ and 3 sham EA groups selected nonspecific acupoints^61^,^78^,^101^. Eight studies utilized pricking sensation to mimic needle sensation and blind participants in control group^20^,^21^,^23^,^52^,^60^,^63^,^67^,^81^. Forty-two studies^19^,^20^,^21^,^22^,^26^,^27^,^28^,^31^,^32^,^33^,^34^,^35^,^37^,^39^,^40^,^42^,^43^,^44^,^46^,^47^,^50^,^51^,^58^,^60^,^61^,^62^,^64^,^65^,^66^,^70^,^71^,^77^,^78^,^79^,^83^,^85^,^89^,^92^,^100^,^104^,^105^,^106^ applied EA at high intensity with maximum tolerance, and seventeen studies^24^,^29^,^54^,^56^,^59^,^80^,^82^,^86^,^87^,^90^,^95^,^98^,^99^,^102^,^107^,^108^,^111^ applied EA with low intensity below pain threshold or at a comfortable level with presence or absence of muscle contractions. The other trials were lacking in details on the intensity of stimulation. The duration of each session ranged from 5 minutes^100^ to 72 hours^96^; the total number of treatment sessions varied from 1^33^,^37^,^38^,^39^,^46^,^47^,^50^,^51^,^52^,^72^,^73^,^77^,^82^,^84^,^86^,^88^,^89^,^94^,^95^,^96^,^97^,^100^,^101^,^102^,^109^ to 72^44^; the total duration of treatment ranged from 5 minutes^100^ to 6 months^111^. Ten studies^27^,^46^,^72^,^76^,^77^,^79^,^89^,^90^,^97^,^102^ did not report the duration of each session. In one study^76^, the total number of treatment session was not mentioned. Eight studies^27^,^46^,^72^,^76^,^77^,^89^,^97^,^102^ did not provide any information on the total duration of treatment. The characteristics of the studies were listed in Table 1.

### Characteristics of sham EA

Ten different types of sham EA methods used in the trials were identified as follows: (1) sham EA type A were used in twenty-six control groups^18^,^19^,^20^,^21^,^22^,^31^,^33^,^37^,^40^,^60^,^66^,^67^,^73^,^74^,^76^,^81^,^94^,^96^,^97^,^102^,^103^,^104^,^106^,^108^,^109^,^110^; (2) sham EA type B were used in seven control groups^23^,^24^,^59^,^65^,^84^,^88^,^105^; (3) sham EA type C were used in seven control groups^28^,^44^,^47^,^49^,^51^,^75^,^82^; (4) sham EA type D were used in six control groups^29^,^43^,^62^,^64^,^72^,^93^; (5) sham EA type F were used in seven control groups^38^,^61^,^71^,^78^,^98^,^99^,^101^; (6) sham EA type L were used in seventeen control groups^26^,^27^,^39^,^43^,^46^,^48^,^50^,^53^,^54^,^61^,^77^,^79^,^85^,^89^,^98^,^99^,^101^; (7) sham EA type M were used in three control groups^42^,^68^,^69^; (8) sham EA type N were used in four control groups^63^,^83^,^90^,^95^; (9) sham EA type O were used in fourteen control groups^30^,^32^,^35^,^41^,^45^,^56^,^57^,^58^,^70^,^80^,^86^,^87^,^91^,^92^; and (10) sham EA type Q were used in ten control groups^25^,^34^,^36^,^38^,^52^,^55^,^63^,^100^,^107^,^111^.

For the needle location, 48 sham EA groups^25^,^26^,^27^,^30^,^32^,^34^,^35^,^36^,^38^,^39^,^41^,^42^,^43^,^45^,^46^,^48^,^50^,^52^,^53^,^54^,^55^,^56^,^57^,^58^,^61^,^63^,^68^,^69^,^70^,^77^,^79^,^80^,^83^,^85^,^86^,^87^,^89^,^90^,^91^,^92^,^95^,^98^,^99^,^100^,^101^,^107^,^111^ chose non-acupoints that were either located away from the therapeutic acupoints with a distance ranging from 1 cm to 40 cm or devised in advance to avoid any known meridian or extra-point. Seven sham EA groups^38^,^61^,^71^,^78^,^98^,^99^,^101^ received nonspecific acupuncture points which were thought to be ineffective for treating the diseases. For the depth of needle insertion, 34 sham EA groups^26^,^27^,^28^,^38^,^39^,^42^,^43^,^44^,^46^,^47^,^48^,^49^,^50^,^51^,^53^,^54^,^61^,^68^,^69^,^71^,^75^,^77^,^78^,^79^,^82^,^85^,^89^,^98^,^99^,^101^ conducted the needle insertion to the same depth as corresponding real EA groups. Twenty-four sham EA groups^29^,^30^,^32^,^35^,^41^,^43^,^45^,^56^,^57^,^58^,^62^,^63^,^64^,^70^,^72^,^80^,^83^,^86^,^87^,^90^,^91^,^92^,^93^,^95^ performed either superficial or subcutaneous needle insertion with depth varying from 0.5 mm to 2 cm, whereas one study^93^ retracted the needle after superficial penetration. The remaining 43 sham EA groups had sham EA without skin penetration. Forty-one out of 43 trials did not apply any needle insertion by using Streitberger needles^21^,^23^,^25^,^31^,^36^,^55^,^59^,^63^,^65^,^66^,^81^, contractible placebo needles with dull tips and tubes^24^,^37^,^52^, placebo needles with blunted tips^19^,^20^,^40^,^60^,^67^,^100^, verum needles fixed by tapes or rings without piercing^18^,^22^,^34^, leading wires alone without needles^33^,^38^,^76^,^97^ and mock laser pen^103^ or electrodes^73^,^74^,^94^,^96^,^102^,^104^,^105^,^106^,^107^,^108^,^109^,^110^,^111^. Two sham EA groups^84^,^88^ did not describe any details on the sham needles. For electrical stimulation, 35 sham EA groups^23^,^24^,^26^,^27^,^38^,^39^,^43^,^46^,^48^,^50^,^53^,^54^,^59^,^61^,^63^,^65^,^71^,^77^,^78^,^79^,^83^,^84^,^85^,^88^,^89^,^90^,^95^,^98^,^99^,^101^,^105^ used electrical stimulation, whereas two sham EA groups stimulated with current just at the beginning of sham procedure. The other sham EA groups^18^,^19^,^20^,^21^,^22^,^25^,^28^,^29^,^30^,^31^,^32^,^33^,^34^,^35^,^36^,^37^,^38^,^40^,^41^,^42^,^43^,^44^,^45^,^47^,^49^,^51^,^52^,^55^,^56^,^57^,^58^,^60^,^62^,^63^,^64^,^66^,^67^,^68^,^69^,^70^,^72^,^73^,^74^,^75^,^76^,^80^,^81^,^82^,^86^,^87^,^91^,^92^,^93^,^94^,^96^,^97^,^100^,^102^,^103^,^104^,^106^,^107^,^108^,^109^,^110^,^111^ did not receive any electrical stimulation through inactivated EA device or disconnected cables. The details of sham EA were described in Table S1.

### Risk of bias assessment

The number of items complied with the criteria varied from 3/8 to 7/8 with the average of 5.2. All 94 studies declared randomization and 63 studies reported the details. Among them, 49 studies^18^,^19^,^22^,^23^,^24^,^25^,^27^,^29^,^35^,^36^,^37^,^38^,^42^,^43^,^44^,^45^,^47^,^49^,^51^,^55^,^59^,^60^,^61^,^62^,^63^,^64^,^65^,^66^,^67^,^70^,^71^,^72^,^81^,^88^,^90^,^91^,^92^,^93^,^96^,^97^,^98^,^99^,^102^,^106^,^107^,^108^,^109^,^110^,^111^ described a computer-generated randomization; 11 studies^32^,^40^,^41^,^50^,^52^,^57^,^69^,^73^,^74^,^85^,^86^ were based on random number Table; 3 studies^83^,^87^,^101^ used the lot. Adequate allocation concealment was found in 43 studies^18^,^19^,^20^,^23^,^25^,^29^,^30^,^35^,^36^,^37^,^43^,^44^,^45^,^55^,^57^,^58^,^60^,^61^,^63^,^64^,^65^,^66^,^67^,^69^,^71^,^72^,^76^,^79^,^81^,^84^,^86^,^88^,^90^,^91^,^93^,^98^,^99^,^105^,^106^,^107^,^109^,^110^,^111^ with sequentially numbered, opaque, sealed envelopes or independent administrator. The remaining 51 studies did not provide the details on allocation concealment. Blinding of participant was described in all 94 studies. Among these, 23 studies^22^,^23^,^25^,^34^,^36^,^45^,^52^,^57^,^59^,^60^,^63^,^65^,^72^,^81^,^88^,^97^,^102^,^103^,^105^,^106^,^107^,^108^,^110^ proved their success of blinding by credibility test, while one study^66^ failed in blinding of participant after testing by statistical analysis. No study mentioned blinding of acupuncturists. Ninety-two out of the 94 studies reported blinding of assessor, whereas one study^54^ did not contain any information on assessor blinding and another study^66^ was sorted as “no” due to its unsuccessful assessor blinding. Eighteen studies^25^,^30^,^45^,^60^,^65^,^69^,^79^,^80^,^91^,^92^,^93^,^97^,^98^,^99^,^103^,^105^,^106^,^107^ conducted intention-to-treat analysis. Seventy-five studies^18^,^19^,^20^,^21^,^22^,^24^,^25^,^26^,^27^,^28^,^29^,^30^,^31^,^32^,^33^,^34^,^35^,^36^,^37^,^38^,^39^,^40^,^41^,^43^,^44^,^46^,^47^,^49^,^50^,^51^,^52^,^53^,^54^,^55^,^56^,^57^,^58^,^59^,^60^,^61^,^62^,^63^,^64^,^65^,^66^,^67^,^68^,^70^,^71^,^72^,^73^,^74^,^75^,^76^,^77^,^78^,^79^,^81^,^82^,^83^,^84^,^85^,^86^,^87^,^88^,^89^,^90^,^91^,^92^,^94^,^95^,^96^,^97^,^100^,^101^,^102^,^103^,^104^,^105^,^108^,^109^,^110^,^111^ were free of incomplete outcome data; eleven studies^23^,^42^,^45^,^48^,^69^,^80^,^93^,^98^,^99^,^106^,^107^ assessed as “no” due to high dropout rate or statistically significant differences between groups in withdrawals from the treatment. The rest were unclear due to lack of information on this aspect. Sixteen studies^36^,^39^,^41^,^42^,^44^,^59^,^63^,^64^,^75^,^79^,^80^,^86^,^91^,^93^,^98^,^99^ were free of selective reporting; one study^65^ was sorted as “no” due to an incomplete outcome measurement report that had been registered in protocol; the others were unclear because such details were not found. Of the 91 trials that provided the information on other bias, 77 studies^18^,^19^,^20^,^21^,^22^,^23^,^24^,^25^,^26^,^27^,^28^,^29^,^30^,^31^,^32^,^33^,^34^,^35^,^36^,^37^,^38^,^39^,^40^,^41^,^42^,^43^,^44^,^45^,^46^,^47^,^48^,^49^,^50^,^51^,^52^,^53^,^54^,^55^,^56^,^60^,^61^,^62^,^63^,^64^,^65^,^66^,^67^,^68^,^70^,^72^,^73^,^74^,^75^,^76^,^79^,^80^,^83^,^84^,^85^,^86^,^87^,^88^,^89^,^91^,^92^,^93^,^94^,^95^,^96^,^97^,^99^,^101^,^103^,^104^,^105^,^109^,^111^ were free of other bias; 14 studies^57^,^58^,^59^,^69^,^78^,^81^,^82^,^90^,^98^,^102^,^106^,^107^,^108^,^110^ were assessed as “no” due to the statistical differences in baseline variables regarding as the most important prognosis. The details on the risk of bias studies were summarized in Table 2.

### Credibility of blinding

Only 24 out of the 94 studies reported the credibility of blinding in participants by conducting the creditability test in six types of sham EA methods. Twenty-three studies^22^,^23^,^25^,^34^,^36^,^45^,^52^,^57^,^59^,^60^,^63^,^65^,^72^,^81^,^88^,^97^,^102^,^103^,^105^,^106^,^107^,^108^,^110^ proved to be successful and one study^66^ proved to be failure. All six types of sham EA methods were claimed to be successful in blinding. They are sham EA type A (10/24 with 1 failure)^22^,^60^,^66^,^81^,^97^,^102^,^103^,^106^,^108^,^110^, type B (5/24)^23^,^59^,^65^,^88^,^105^, type Q (5/24)^25^,^34^,^36^,^52^,^107^, sham EA type O (2/24)^45^,^57^, sham EA type D (1/24)^72^, and sham EA type N (1/24)^63^.

### Efficacy results of the included studies

All 94 studies involving 105 comparisons of real and sham EA groups provided the information for between-groups analyses. Among them, 59 real EA groups^18^,^19^,^21^,^22^,^24^,^28^,^30^,^33^,^34^,^35^,^37^,^38^,^40^,^41^,^42^,^43^,^44^,^48^,^49^,^51^,^53^,^54^,^57^,^59^,^60^,^61^,^62^,^63^,^64^,^65^,^69^,^70^,^71^,^72^,^73^,^75^,^77^,^78^,^79^,^83^,^84^,^86^,^87^,^88^,^90^,^91^,^92^,^94^,^95^,^97^,^99^,^100^,^104^,^108^ reported significant superiority over corresponding sham EA groups; forty-three real EA groups were not statistically better than sham EA groups; one study^74^ showed that sham EA group was superior to the real EA group; the remaining two studies^50^,^111^ lacked original data for between-groups analyses and were stated as “T > C?”. The efficacy results of the studies are listed in Table 1 and summarized in Table 3 according to different types of sham EA methods and EA indications.

Compared with sham EA controls, EA therapy in about 56.2% (59/105 comparisons) of comparisons reported the specific effect. Correspondingly, the real EA was superior to sham EA for type N (75%, 3/4 comparisons), type F (71.4%, 5/7 comparisons), type D (66.7%, 4/6 comparisons) and type M (66.7%, 2/3 comparisons). The lowest percentage of positive efficacy result was 44.4% (8/18 comparisons) in sham EA type L. The positive rate of efficacy for the three most often used sham EA methods were 50% (13/26 comparisons) for sham EA type A, 44.4% (8/18 comparisons) for sham EA type L and 64.3% (9/14 comparisons) for sham EA type O.

The type of sham EA methods varied across different EA indications. The sham EA type A was most commonly used in RCTs for pain, anesthesia and osteoarthritis. The sham EA type D and sham EA type Q were applied mainly in stroke studies. The sham EA type B was commonly applied to RCTs on depression. The sham EA type L and sham EA type O were commonly performed in trials on obesity. The sham EA type F and sham EA type L were commonly used in studies on primary dysmenorrhea.

## Discussion

To our knowledge, this is the first systematic analysis to address sham EA methods in RCTs. The numbers of publications and sham EA methods have been increasing every decade. We summarized seventeen kinds of sham EA methods according to three aspects as needle location, depth of needle insertion and electrical stimulation, whereas only ten types of sham EA methods were identified from 94 included RCTs involving 6134 participants. The three predominant types of sham EA methods used were sham EA type A, type L and type O ordinally. Only 24 out of 94 trials reported credibility test with the results of 23 success and 1 failure using six types of sham EA methods mainly as follows: sham EA type A (10/24 with 1 failure), type B (5/24) and type Q (5/24). The remaining 3 sham EA methods were only tested in 4 trials. About 56.2% of comparisons provided the evidence of specific effect of EA therapy, and the four types of Sham EA controls with highest positive rate of efficacy result were type N (75%, 3/4 comparisons), type F (71.4%, 5/7 comparisons), type D (66.7%, 4/6 comparisons) and type M (66.7%, 2/3 comparisons) ordinally. However, all types of Sham EA controls were used in a small number of trials. Thus, the evidence was insufficient to recommend any type of sham EA control despite of the high positive rate. The sham EA control was frequently used in RCTs for pain, anesthesia, stroke, depression, obesity and primary dysmenorrheal/menstrual pain, suggesting that these diseases are particularly worthy of further EA RCTs.

The ideal design of sham acupuncture method remains methodologically challenging^112^. Consequently, a great variety of emerging sham acupuncture methods have found their ways into present RCTs by using non-traditional Chinese medicine acupoint^26^,^27^,^113^, no or superficial penetration^29^ and no or suboptimal stimulation^28^. The sham procedures in acupuncture RCTs were previously summarized by He *et al*.^9^ as seven types. A previous review by Dincer *et al*.^8^ reported the classification of sham acupuncture as sham type I~V based on three respects as needle location, insertion and stimulation. In the present study, we focused on the sham EA methods according to three aspects as needle location, depth of needle insertion and electrical stimulation, and summarized seventeen types of sham EA methods. Ten types of sham EA methods were actually used in the included RCTs.

The main purpose of RCTs on EA is to evaluate its specific effect. An optimal sham acupuncture technique must be biologically inactive and psychologically credible^114^. A lot of practice has been done to make the sham components of EA less perceptually and operationally distinguishable from real EA intervention for the purpose of keeping the blinding status of the participants. Streitberger needles, blunted needles and verum needles were frequently used with foam, tape or tube for hiding acupuncture loci from subjects^18^,^19^,^21^,^22^. Furthermore, a pricking sensation was elicited by dull tips for concealing the perceptual differences^20^,^21^,^23^,^52^,^60^,^63^,^67^,^81^. The sham EA device was often accompanied by indicator light or with sound signals for confusing the participants^18^,^22^,^37^. In the present study, six types of sham EA method were tested as concealable control in terms of blinding of participants.

The top three types of sham EA methods used were sham EA type A, type L and type O. The most frequently applied sham EA method was type A, accounting for a popular belief in its inertness based on its absence of key EA components as needle stimulation and electrical stimulation as well as its indistinguishable manipulation on same therapeutic acupoints. In the present study, the validation of credible participant blinding of this sham EA type was reported by most credibility tests. The debate emerged over the past decades over the inertness of non-penetrating procedure since the slight acupressure effects and physiological activity might be evoked by the tactile stimulation from blunt needle tips even without skin penetration^112^,^115^. Takayama *et al*.^116^ argued that non-penetrating placebo needle is at least clinically inert for pain alleviation based on their cross-over study reporting no analgesic effects of the skin-touch placebo needle over that of the no-touch placebo or that of the no-treatment control. However, conclusive evidence are out of our awareness up to now whether non-penetrating but skin-touch placebo needle plays a specific therapeutic role in other medical condition. Thus, the sham EA type A may be an promising candidate control for further RCTs on analgesic effects of EA and relative further researches are called for in aspect of any other conditions.

Sham EA type Q is deemed to be the most inert type of sham EA control because it avoids all therapeutic components, which also probably makes this sham method perceptually and operationally distinguishable from real EA intervention and to some extent results in problematic credibility of blinding in participant. In the present study, the credibility of participant blinding of this sham EA type was endorsed by five studies with credibility test^25^,^34^,^36^,^52^,^107^. However, mechanical non-penetration can evoke brain responses in healthy subjects. Thus, controversy raised regarding whether this type of sham EA method is physiologically inert control^117^. Moreover, four-fifth of the studies were conducted on acupuncture naïve participants. There is a possibility that previous experience of acupuncture treatments might have an impact on present perception of verum and sham EA intervention, which should be rigorously controlled in EA RCTs to avoid bias from unblinding. With the informed consent lack of explicit information on the sham method, debates emerged over ethical acceptability of the study.

The second commonly used sham EA method was type L. It was found that the differential effects of real EA and sham EA, which were attributed to point location, was not consistent across studies and conditions within this sham EA type, suggesting that EA on non-acupoints might be efficacious as EA on therapeutic acupoints. Furthermore, the improvements from baseline were also observed by Sahin *et al*.^26^, Li *et al*.^46^ and Yu *et al*.^43^ The similar findings were previously presented by Moffet *et al*.^118^ showing that sham acupuncture at non-acupoints was as efficacious as true acupuncture. It seemed that in the above studies the specificity of acupoints does not exit and to some extent were in violation of traditional acupuncture theories. Li *et al*.^46^ stated that the specificity of acupoints was not present in EA treatment. However, Wang *et al*.^79^ argued for the specificity of acupoints in EA treatment based on the better effects of EA at acupoints than that at non-acupoints on certain clinical outcomes. From the heterogeneous evidence of acupoint specificity, no definitive conclusion could be drawn based on the paucity of available high-quality clinical trials^119^. The main issue in this sham EA type might lie in the accurate identification of non-acupoint rather than a rough location nearby traditional acupoint that might be responsible for specific effects, which rises the challenge of conducting appropriate sham control in EA clinical trials especially in the presence or absence of the mechanism of acupoint specificity and the consistency in finding actual point across the different practitioners. Nevertheless, it is unclear whether this type is a concealable control for participant blinding since this sham EA tested credibility in the present study. Therefore, it demonstrates that sham EA type L might not be adequately controlled from inert or concealable perspective only if the mechanisms of acupoints were explicitly explored or the validation of so-called non-acupoint was verified by further researches. Cautions should be taken for eliminating bias from this sham EA control type.

The third commonly used sham EA was type O. During the procedure, the shallow insertion was applied to simulate deep skin penetration and to ensure the blinding of participant. In the present study, the validation of participant blinding of this sham EA type was endorsed by two studies^45^,^57^. As for the issue of inertness, a few studies reported that sham EA control improved baseline in certain clinical parameters compared with conventional group^32^,^46^,^56^,^57^,^58^. Moffet *et al*.^118^ stated that shallow needling at non-acupoints might be as efficacious as real acupuncture. Lund *et al*.^120^ reported that minimal acupuncture based on superficial insertion was not a valid control from a physiological perspective. Hróbjartsson *et al*.^121^ held the view that sham EA type was not inert control from Chinese medicine perspective. A sham EA procedure with superficial needling at non-acupoints might have subliminal effects since the locations of points was nearby true acupoints or myotome. Moreover, it is likely that the superficial insertion was not consistently applied since the needling depth varied across differentacupoints on different body parts and the relatively deep insertions might be conducted for taking the weight of the attached electrodes. Thus, this sham EA type may be concealable control but far from inert control in RCTs for EA, unless the extent to which the sham procedure could be regarded as physiologically inert has been clarified.

In the present study, six types of sham EA method were reported as successful in blinding. However, further investigations are needed for confirmation, since half of the tested types of sham EA controls were reported in a small number of trials. It should be noted that studies included did not provide sufficient evidence of blinding in acupuncturist. Vase *et al*.^122^ stated that it was hard to get acupuncture intervention fully double-blinded. Although non-penetrating needle was previously reported as potential sham control to mask both participants and practitioners in acupuncture research^123^,^124^,^125^, a previous review demonstrated that the acupuncture intervention was not fully double-blinded^122^. New strategies should be implemented for the development of double-blind sham EA control in terms of both participants and acupuncturists.

RCTs are generally recognized as the gold standard for the efficacy of clinical interventions by excluding the non-specific effect via a placebo control^126^. However, one study reported that the effect of EA therapy was merely the non-specific effect^63^. In the present study, the number of real EA group with superiority to, no difference from and inferiority to corresponding sham EA group was 59, 43 and 1, respectively. Thus, more than half comparisons demonstrated that EA therapy existed specific effects. Within all types of sham EA methods, the highest effective rate were type N (75% 3/4 comparisons), type F (71.4%, 5/7 comparisons), type D (66.7%, 4/6 comparisons) and type M (66.7%, 2/3 comparisons) successively. Considering the small number of included studies within corresponding sham EA type, the evidence are still insufficient to recommend any type of sham EA control despite of the high positive rate.

In the present study, 43/105 comparisons reported that EA has no specific effects compared with sham EA controls^20^,^23^,^24^,^25^,^26^,^27^,^29^,^31^,^32^,^36^,^39^,^43^,^44^,^45^,^46^,^47^,^52^,^55^,^56^,^58^,^63^,^66^,^67^,^68^,^76^,^80^,^81^,^82^,^85^,^89^,^93^,^96^,^98^,^101^,^102^,^103^,^105^,^106^,^107^,^109^,^110^. For a reason, the extent to which the individual component of EA intervention plays its therapeutic influences on the final outcomes is not clear during clinical treatment^7^. The debate consequently emerges regarding the therapeutic inactivity of sham EA control which is partially comprised of real EA components, such as suboptimal manual or electrical stimulation^110^. The probability in the specific effects of EA may be reduced by the potential activity produced by sham EA control. On the other hand, EA is a complex intervention method. Its therapeutic effects consist of specific effects from needling and stimulation components as well as moderately large nonspecific effects, which means that the efficacy results of RCTs for EA are more likely to be influenced by a variety of factors, such as patient/practitioner interaction and patient expectations^127^,^128^. In the clinical use, EA may be more effective than manual acupuncture in some situations such as when strong, continued stimulation is required, and when treating pain, anesthesia, stroke, depression, obesity and primary dysmenorrhea/menstrual pain, suggesting that further RCTs with appropriate sham EA control are in need to verify the specific effects on above conditions.

There are several weaknesses in the present study. Firstly, the search and screen procedure were limited to randomized, parallel-controlled trials published in English. Thus, those trials with cross-over design or published in other than English language were omitted. Secondly, with the aim of evaluating the sham method in RCTs on EA, a generous criterion was established to select eligible studies. Therefore, it was not easy to examine the specific effect of EA by data synthesis from different outcomes and indications because of the heterogeneity of trials. Finally, the reported credibility test addressed blinding effects in participant rather than in both participant and acupuncturist. The credibility tests were not reported in all studies and the number of studies using sham EA types was small, and therefore the conclusion should be interpreted with cautions.

## Conclusion

Ten types of sham EA methods were identified based on our scheme classification. Generally, sham EA type A, type L and type O were frequently used. Yet, further clinical trials are recommended to maintain standard methodology of concealable and inert placebo EA techniques. Only 24 out of 94 trials were reported as positive credibility test in six types of sham EA methods, where sham EA type A, type B and type Q were highly practiced. It is worthy to study further about the importance of concealable sham EA types. EA therapy in approximately, 56.2% of comparisons provided the specific effects. The four types of sham EA (N, F, D and M respectively) represented the highest positive rate of efficacy results. However, progressive evidences on specific effects are mandatory. The sham EA control was observed frequently in pain, anesthesia, stroke, depression, obesity and primary dysmenorrhea RCTs. Also, broader studies in these predominant diseases are advised.

## Additional Information

**How to cite this article**: Chen, Z. *et al*. Sham Electroacupuncture Methods in Randomized Controlled Trials. *Sci. Rep.* **7**, 40837; doi: 10.1038/srep40837 (2017).

**Publisher's note:** Springer Nature remains neutral with regard to jurisdictional claims in published maps and institutional affiliations.

## Supplementary Material

Supplementary Information

## Figures and Tables

**Table 1:** Characteristics of 94 included studies.

Reference (author, year and country)	Diseases	No. of acupoint (T/C)	Sample size (T/C)	dropout (T/C)	Primary outcome measures	Difference between groups	Characteristic of sham electro-acupuncture (EA) methods			
							needle location	degree of needle insertion	electrical stimulation	The type of sham EA method
Ntritsou *et al*. 2014 USA^18^	Pain	4/4	37/38	5(2/3)	NRS and SF_MPQ scores	T > C	therapeutic acupoints	no penetration	no electrical stimulation	Sham EA type A
Chu *et al*. 2012 Hong Kong^19^	Pain perception in Irritable Bowel Syndrome	6/6	15/15	0(0/0)	FMRI	T > C	therapeutic acupoints	no penetration	no electrical stimulation	Sham EA type A
Wang *et al*. 2010 Denmark^20^	Tinnitus	4/14	20/20	9(4/5)	The frequency of tinnitus occurrence and the tinnitus loudness	ND	therapeutic acupoints	no penetration	no electrical stimulation	Sham EA type A
Zyloney *et al*. 2010 USA^21^	Pain	2/2	N/N	29(N/N)	FMRI and MASS ratings	T > C	therapeutic acupoints	no penetration	no electrical stimulation	Sham EA type A
Jubb *et al*. 2008 UK^22^	Osteoarthritis	6/6	34/34	4(2/2)	WOMAC pain score	T > C	therapeutic acupoints	no penetration	no electrical stimulation	Sham EA type A
Chung *et al*. 2012 Hong Kong^23^	Postpartum Depression	18/18	10/10	6(5/1)	EPDS, HADS, HDRS_17_ and CGI scores	ND	therapeutic acupoints	no penetration	electrical stimulation	Sham EA type B
Barlas *et al*. 2006 UK^24^ 1	Pain	4/4	12/12	N(N/N)	Pressure pain threshold	T > C	therapeutic acupoints	no penetration	electrical stimulation	Sham EA type B
Barlas *et al*. 2006 UK^24^ 2	Pain	4/4	12/12	N(N/N)	Pressure pain threshold	ND	therapeutic acupoints	no penetration	electrical stimulation	Sham EA type B
Wayne *et al*. 2005 US^25^	Stroke Rehabilitation	14–22/2–3	16/17	9(3/6)	FMA score	ND	non-acupuncture points	no penetration	no electrical stimulation	Sham EA type Q
Sahin *et al*. 2010 Turkey^26^	Pain	14/14	15/16	2(2/0)	VAS scores	ND	non-acupuncture points	the same depth	electrical stimulation	Sham EA type L
Fanti *et al*. 2003 Italy^27^	Pain	10/10	10/10	0(0/0)	VAS scores	ND	non-acupuncture points	the same depth	Electrical stimulation	Sham EA type L
Hsing *et al*. 2012 Brazil^28^	Stroke recovery	11–14/11–14	31/31	0(0/0)	NIHSS, Barthel Index and modified Rankin scales scores	T > C	therapeutic acupoints	the same depth	no electrical stimulation	Sham EA type C
Gosman-Hedström *et al*. 1998 Sweden^29^	Stroke	4/4	37/34	3(2/1)	The neurological score and the Barthel and Sunnaas index scores	ND	therapeutic acupoints	superficial penetration	no electrical stimulation	Sham EA type D
Jing *et al*. 2009 China^30^	Auditory hallucination	6/6	30/30	7(4/3)	The Psychotic Symptom Rating Scales and Auditory Hallucination Subscale total score	T > C	non-acupuncture points	superficial penetration	no electrical stimulation	Sham EA type O
Kong *et al*. 2009 USA^31^	Pain	2/2	N/N	29(N/N)	Gracely Sensory and Affective scales scores and fMRI	ND	therapeutic acupoints	no penetration	no electrical stimulation	Sham EA type A
Darbandi *et al*. 2013 Iran^32^	Obesity	4/4	47/47	8(5/3)	BW, BMI and BFM	ND	non-acupuncture points	superficial penetration	no electrical stimulation	Sham EA type O
Yang *et al*. 2012 China^33^	Pain	12/12	40/40	0(0/0)	the consumption of sevoflurane and the recovery profile	T > C	therapeutic acupoints	no penetration	no electrical stimulation	Sham EA type A
Liu *et al*. 2013 China^34^	Post-stroke detrusor overactivity	10/10	35/36	5(2/3)	maximum cystometric capacity and bladder compliance	T > C	non-acupuncture points	no penetration	no electrical stimulation	Sham EA type Q
Zhang *et al*. 2014 China^35^	Postoperative ileus	2/2	20/20	1(1/0)	Time of the first bowel sounds and passage of flatus	T > C	non-acupuncture points	superficial penetration	no electrical stimulation	Sham EA type O
Mao *et al*. 2014 USA^36^	Aromatase Inhibitor-Related Arthralgia	4/4	22/22	6(3/3)	BPI	ND	non-acupuncture points	no penetration	no electrical stimulation	Sham EA type Q
Leung *et al*. 2011 Hong Kong^37^	Pain	3/3	20/20	0(0/0)	the maximal tolerable pressure, VAS score and beta-endorphin level	T > C	therapeutic acupoints	no penetration	no electrical stimulation	Sham EA type A
Rusy *et al*. 2002 USA^38^ a	Postoperative Nausea and Vomiting	1/1	40/40	0(0/0)	occurrence of nausea and vomiting, and use of antiemetic rescue medication	T > C	nonspecific acupuncture points	the same depth	electrical stimulation	Sham EA type F
Rusy *et al*. 2002 USA^38^ b	Postoperative Nausea and Vomiting	1/1	40/40	0(0/0)	occurrence of nausea and vomiting, and use of antiemetic rescue medication	T > C	non-acupuncture points	no penetration	no electrical stimulation	Sham EA type Q
Yang *et al*. 2014 China^39^	Migraine	3/3	10/10	0(0/0)	VA S scores	ND	non-acupuncture points	the same depth	Electrical stimulation	Sham EA type L
Chen *et al*. 2013 China^40^	Pancreatic cancer pain	10/10	30/30	1(0/1)	NRS	T > C	therapeutic acupoints	no penetration	no electrical stimulation	Sham EA type A
Quispe-Cabanillas *et al*. 2012 Brazi^41^	Multiple sclerosis	9/9	16/15	0(0/0)	EDSS, pain VASscore and quality of life FAMS	T > C	non-acupuncture points	superficial penetration	no electrical stimulation	Sham EA type O
Aranha *et al*. 2015 Brazil^42^	Pain	7–8/7–8	24/23	17(7/10)	VAS scores and cervical movements	T > C	non-acupuncture points	the same depth	no electrical stimulation	Sham EA type M
Yu *et al*. 2013 Hong Kong^43^ a		2/2	12/12	0(0/0)	C-MMASS and HR, MAP	ND	non-acupuncture points	the same depth	Electrical stimulation	Sham EA type L
Yu *et al*. 2013 Hong Kong^43^ b		2/2	12/12	0(0/0)	C-MMASS and HR, MAP	T > C	therapeutic acupoints	superficial penetration	no electrical stimulation	Sham EA type D
Li *et al*. 2010 Chin^44^	Attention deficit hyperactivity disorder	15–16/15–16	92/88	10(6/4)	relapse rate	T > C	therapeutic acupoints	the same depth	no electrical stimulation	Sham EA type C
Zheng *et al*. 2007 Australia^45^	Chronic pain	4/4	17/18	12(8/4)	the dosage reduction of OLM, the incidence of side effect, and VAS score	ND	non-acupuncture points	superficial penetration	no electrical stimulation	Sham EA type O
Li *et al*. 2013 China^46^	General anesthesia	10/6	9/10	0(0/0)	the levels of TNF-α, IL-8 and IL-10	ND	non-acupuncture points	the same depth	Electrical stimulation	Sham EA type L
Lin *et al*. 2002 Taiwan^47^ 1	Postoperative pain	2/2	25/25	0(0/0)	VAS score	ND	therapeutic acupoints	the same depth	no electrical stimulation	Sham EA type C
Lin *et al*. 2002 Taiwan^47^ 2	Postoperative pain	2/2	25/25	0(0/0)	VAS score	ND	therapeutic acupoints	the same depth	no electrical stimulation	Sham EA type C
Chen *et al*. 2013 Taiwan^48^	Constipation	6/6	30/30	30 (16/14)	the defecation rate	T > C	non-acupuncture points	the same depth	Electrical stimulation	Sham EA type L
Schukro *et al*. 2014 Austria^49^	Obesity	3/3	28/28	14(7/7)	the relative reduction of body weight	T > C	therapeutic acupoints	the same depth	no electrical stimulation	Sham EA type C
Yu *et al*. 2014 China^50^	General anesthesia	2/2	20/20	0(0/0)	The serum cortisol and ACTH	T > C ?	non-acupuncture points	the same depth	Electrical stimulation	Sham EA type L
Xie *et al*. 2014 China^51^	Postoperative pain	2/2	20/20	0(0/0)	VAS score, Total Doses of Sufentanil and Dezocine	T > C	therapeutic acupoints	the same depth	no electrical stimulation	Sham EA type C
Sim *et al*. 2002 Singapore^52^	Intraoperative pain	N/N	30/30	0(0/0)	The total intraoperative usage of alfentanil, The total morphine consumption and VAS score	ND	non-acupuncture points	no penetration	no electrical stimulation	Sham EA type Q
Song *et al*. 2009 China^53^	Depression	2/2	31/32	10(3/7)	HDRS and CGI	T > C	non-acupuncture points	the same depth	Electrical stimulation	Sham EA type L
Shafshak 1995 Egypt^54^ 1	Obesity	2/2	10/10	N(N/N)	the success rate of going on the diet trigmen	T > C	non-acupuncture points	the same depth	Electrical stimulation	Sham EA type L
Shafshak 1995 Egypt^54^ 2	Obesity	2/2	10/10	N(N/N)	the success rate of going on the diet trigmen	T > C	non-acupuncture points	the same depth	Electrical stimulation	Sham EA type L
Franasiak *et al*. 2012 USA^55^	Polycystic Ovary Syndrome	8/8	46/50	16(9/7)	Serum LH and FSH.The monthly rates of ovulation	ND	non-acupuncture points	no penetration	no electrical stimulation	Sham EA type Q
Naslund *et al*. 2002 Sweden^56^	Idiopathic anterior knee pain	6/6	30/28	1(0/1)	one leg vertical jump, functional score, daily VAS recording and skin temperature	ND	non-acupuncture points	superficial penetration	no electrical stimulation	Sham EA type O
Shen *et al*. 2000 US^57^	Chemotherapy–Induced Emesis	4/4	37/33	1(1/1)	Total number of emesis episodes occurring	T > C	non-acupuncture points	superficial penetration	no electrical stimulation	Sham EA type O
Wyon *et al*. 2004 Sweden^58^	Hot flushes in postmenopausal women.	6/6	15/15	7(4/3)	The number of flushes/24 h	ND	non-acupuncture points	superficial penetration	no electrical stimulation	Sham EA type O
Zhang *et al*. 2012 Hong Kong^59^	Depression	12/12	38/35	10(7/3)	score of HAMD-17 and SDS	T > C	therapeutic acupoints	no penetration	electrical stimulation	Sham EA type B
Zheng *et al*. 2010 Australia^60^	Pain	2/2	12/12	0(0/0)	SPT and TST	T > C	therapeutic acupoints	no penetration	no electrical stimulation	Sham EA type A
Ma *et al*. 2010 China^61^ a	Menstrual Pain	2/2	13/14	1(1/0)	VAS scores	T > C	nonspecific acupuncture points	the same depth	electrical stimulation	Sham EA type F
Ma *et al*. 2010 China^61^ b	Menstrual Pain	2/2	13/12	1(1/0)	VA S scores	T > C	non-acupuncture points	the same depth	Electrical stimulation	Sham EA type L
Wang *et al*. 2014 Taiwan^62^	Chronic stroke	4/4	10/10	5(1/4)	R1, R2 and R2–R1	T > C	therapeutic acupoint	superficial penetration	No electrical stimulation	Sham EA type D
Yeung *et al*. 2011 Hong Kong^63^ a	Insomnia	8/8	26/26	7(4/3)	ISI and PSQI	ND	non-acupuncture points	superficial penetration	electrical stimulation	Sham EA type N
Yeung *et al*. 2011 Hong Kong^63^ b	Insomnia	8/8	26/26	7(4/3)	ISI and PSQI	T > C	non-acupuncture points	No penetration	No electrical stimulation	Sham EA type Q
Chan *et al*. 2014 Taiwan^64^	Heroin Addicts	4/4	30/30	2(1/1)	the daily consumption of methadone	T > C	therapeutic acupoint	superficial penetration	no electrical stimulation	Sham EA type D
Man *et al*. 2014 Hong Kong^65^	Post-stroke depression	20/20	23/20	10(4/6)	HAMD-17 and CGI-S	T > C	therapeutic acupoint	No penetration	electrical stimulation	Sham EA type B
Oh *et al*. 2013 Australia^66^	Pain	16/12	16/16	3(2/1)	WOMAC, BPI-SF and FACT-G instrument	ND	therapeutic acupoint	No penetration	No electrical stimulation	Sham EA type A
Wong *et al*. 2006 Hong Kong^67^	Pain	4/4	13/14	2(0/2)	VAS pain score	ND	therapeutic acupoint	No penetration	No electrical stimulation	Sham EA type A
Song *et al*. 2007 USA^68^	Depression	2/2	N/N	N(N/N)	24-item HAMD and the level of G protein α subtypes in the platelet membrane	ND	non-acupuncture points	the same depth	no electrical stimulation	Sham EA type M
Cameron *et al*. 2011 Australia^69^	Whiplash-associated Disorders	8/8	64/60	8(0/8)	VAS	T > C	non-acupuncture points	the same depth	no electrical stimulation	Sham EA type M
Darbandi *et al*. 2014 Iran^70^	Obesity	4/4	20/20	0(0/0)	BMI, TFM, WC and HC	T > C	non-acupuncture points	superficial penetration	no electrical stimulation	Sham EA type O
Wei *et al*. 2008 China^71^	Postpartum Insufficient Lactation	2/2	46/46	0(0/0)	total therapeutic effect, 24-hour milk secretion quantity, prolactin level	T > C	nonspecific acupuncture points	the same depth	electrical stimulation	Sham EA type F
Wang *et al*. 2007 USA^72^	Pain	4/4	29/27	0(0/0)	intraprocedural alfentanil consumption, VAS score	T > C	therapeutic acupoint	superficial penetration	no electrical stimulation	Sham EA type D
Kvorning *et al*. 2003 Sweden^73^	Anaesthesia	12/12	23/23	1(1/0)	Physiological reactions to skin incision	T > C	therapeutic acupoint	No penetration	no electrical stimulation	Sham EA type A
Kvorning *et al*. 2003 Sweden^74^	Anaesthesia	6/6	23/23	0(0/0)	The minimal alveolar concentration of sevoflurane	T < C	therapeutic acupoint	No penetration	no electrical stimulation	Sham EA type A
Yang *et al*. 2010 China^75^	Cardiac ischemia-reperfusion injury	6/6	30/30	0(0/0)	levels of serum cardiac troponin I	T > C	therapeutic acupoint	the same depth	no electrical stimulation	Sham EA type C
Sahmeddini *et al*. 2010 Iran^76^	Perioperative Pain	4/4	45/45	0(0/0)	score on VAS-100	ND	therapeutic acupoint	no penetration	no electrical stimulation	Sham EA type A
Wang *et al*. 2007 China^77^		1/1	9/5	3(3/0)	BOLD fMRI	T > C	non-acupuncture points	the same depth	electrical stimulation	Sham EA type L
Jia *et al*. 2009 China^78^	Migraine	2/2	138/138	1(0/1)	VAS score and the plasma 5-HT level	T > C	nonspecific acupuncture points	the same depth	electrical stimulation	Sham EA type F
Wang *et al*. 2013 China^79^	Benign prostate hyperplasia	2/2	50/50	23(9/14)	IPSS	T > C	non-acupuncture points	the same depth	electrical stimulation	Sham EA type L
Andreescu *et al*. 2011 Canada^80^	Depression	2/2	28/29	11(4/7)	HDRS score	ND	non-acupuncture points	superficial penetration	No electrical stimulation	Sham EA type O
Yeung *et al*. 2009 Hong Kong^81^	Insomnia	8/8	30/30	3(1/2)	ISI	ND	therapeutic acupoint	no penetration	no electrical stimulation	Sham EA type A
Chen *et al*. 2014 Taiwan^82^	Pain	1/1	25/24	0(0/0)	VAS scores and the dosage of opium derivative analgesic	ND	therapeutic acupoint	the same depth	no electrical stimulation	Sham EA type C
Wang *et al*. 2008 China^83^	Diabetic Gastroparesis	4/4	11/12	4(2/2)	GCSI score	T > C	non-acupuncture points	superficial penetration	electrical stimulation	Sham EA type N
Meissner *et al*. 2004 Germany^84^	Pain	6/6	8/8	0(0/0)	SEPs	T > C	therapeutic acupoint	no penetration	electrical stimulation	Sham EA type B
Zhou *et al*. 2012 China^85^		2/2	11/11	0(0/0)	MVC strength	ND	non-acupuncture points	the same depth	electrical stimulation	Sham EA type L
Yeh *et al*. 2012 Taiwan^86^	Shivering during regional anesthesia	4/4	40/40	0(0/0)	Shivering score and tympanic temperature	T > C	non-acupuncture points	superficial penetration	no electrical stimulation	Sham EA type O
Yu *et al*. 2011 Taiwan^87^	Benign Prostate Hyperplasia	6/6	21/21	5(3/2)	The change of the maximum flow rate, average flow rate, void volume	T > C	non-acupuncture points	superficial penetration	no electrical stimulation	Sham EA type O
Ma *et al*. 2011 China^88^	Pain	2/2	116/117	0(0/0)	VAS score	T > C	therapeutic acupoint	no penetration	no electrical stimulation	Sham EA type B
Li *et al*. 2013 China^89^	Intraoperative immunosuppression	10/6	19/19	0(0/0)	the levels of TNFα, IL-8, IL-10, IgM, IgA, IgG and full blood count	ND	non-acupuncture points	the same depth	electrical stimulation	Sham EA type L
Deluze *et al*. 1992 Switzerland^90^	Fibromyalgia	4-10/4	36/34	15(8/7)	Pain threshold, number of analgesic tablets used, VAS score	T > C	non-acupuncture points	superficial penetration	electrical stimulation	Sham EA type N
Ng *et al*. 2013 Hong Kong^91^	Postoperative ileus	4/4	55/55	0(0/0)	Time to defecation	T > C	non-acupuncture points	superficial penetration	no electrical stimulation	Sham EA type O
Lee and Lee 2009 Republic of Korea^92^	Chronic Prostatitis / Chronic Pelvic Pain Syndrome	6/6	13/13	5(2/3)	NIH-CPSI	T > C	Non-acupuncture points	superficial penetration	no electrical stimulation	Sham EA type O
Tam *et al*. 2007 Hong Kong^93^	Rheumatoid arthritis	6/6	12/12	5(0/5)	VAS	ND	Therapeutic acupoint	Superficial penetration	No electrical stimulation	Sham EA type D
Kvorning and Akeson 2010 Sweden^94^	Anaesthesia	12/12	22/23	0(0/0)	Plasma levels of adrenaline	T > C	Therapeutic acupoint	No penetration	No electrical stimulation	Sham EA type A
Waite and Clough 1998 UK^95^	Smoking cessation	2/2	40/38	0(0/0)	Biochemically validated total cessation of smoking	T > C	Non-acupuncture points	superficial penetration	Electrical stimulation	Sham EA type N
Holzer *et al*. 2011 Austria^96^	Postoperative pain	3/3	20/20	0(0/0)	VAS scores and the consumption of piritramide	ND	Therapeutic acupoint	No penetration	No electrical stimulation	Sham EA type A
Sator-Katzenschlager *et al*. 2006 Austria^97^	Perioperative pain	3/3	32/30	1(0/1)	VAS scores, adverse event and analgesic drug consumption	T > C	Therapeutic acupoint	No penetration	No electrical stimulation	Sham EA type A
Liu *et al*. 2011 China^98^ a	Primary dysmenorrhea	1/1	50/50	5(3/2)	VAS scores	ND	Nonspecific acupuncture points	The same depth	Electrical stimulation	Sham EA type F
Liu *et al*. 2011 China^98^ b	Primary dysmenorrhea	1/1	50/46	6(3/3)	VAS scores	ND	Non-acupuncture points	The same depth	Electrical stimulation	Sham EA type L
Liu *et al*. 2014 China^99^ a	Primary dysmenorrhea	2/2	167/167	6(4/2)	VAS scores	T > C	Nonspecific acupuncture points	The same depth	Electrical stimulation	Sham EA type F
Liu *et al*. 2014 China^99^ b	Primary dysmenorrhea	2/2	167/167	6(4/2)	VAS scores	T > C	Non-acupuncture points	The same depth	Electrical stimulation	Sham EA type L
Maeda *et al*. 2013 USA^100^ 1	Carpal tunnel syndrome	2/2	22/19	0(0/0)	functional MRI, VAS scores	T > C	Non-acupuncture points	No penetration	No electrical stimulation	Sham EA type F
Maeda *et al*. 2013 USA^100^ 2	Carpal tunnel syndrome	2/2	18/19	0(0/0)	functional MRI, VAS scores	T > C	Non-acupuncture points	No penetration	No electrical stimulation	Sham EA type F
Shi *et al*. 2011 China^101^ a	Primary dysmenorrhea	1/1	10/10	0(0/0)	VAS scores, The plasma PGE_2_, PGF_2a_, TXB_2_, and 6-keto PGF_1a_ levels	ND	Nonspecific acupuncture points	The same depth	Electrical stimulation	Sham EA type F
Shi *et al*. 2011 China^101^ b	Primary dysmenorrhea	1/1	10/10	0(0/0)	VAS scores, The plasma PGE2, PGF2a, TXB2, and 6-keto PGF1a levels	ND	Non-acupuncture points	The same depth	Electrical stimulation	Sham EA type L
Dias *et al*. 2010 Brazil^102^	Local anaesthesia	8/8	16/17	0(0/0)	VAS scores	ND	Therapeutic acupoint	No penetration	No electrical stimulation	Sham EA type A
Zhang *et al*. 2013 Hong Kong^103^	Chronic neck Pain	5/5	103/103	46(19/27)	NPQ scores	ND	Therapeutic acupoint	No penetration	No electrical stimulation	Sham EA type A
Sangdee *et al*. 2002 Thailand^104^	Osteoarthritis of the knee	4/4	48/47	4(2/2)	VAS score, and Lequesne’s functional index	T > C	Therapeutic acupoint	No penetration	No electrical stimulation	Sham EA type A
Johansson *et al*. 2001 Sweden^105^	Stroke rehabilitation	9–10/4	48/51	20(11/9)	The scores of Barthel Index, the Rivermead Mobility Index and NHP, the time needed to walk 10 meters	ND	Therapeutic acupoint	No penetration	Electrical stimulation	Sham EA type B
Hopwood *et al*. 2008 UK^106^	Stroke recovery	8–10/6	57/48	13(10/3)	The scores of Barthel Index	ND	Therapeutic acupoint	No penetration	No electrical stimulation	Sham EA type A
White *et al*. 1998 England^107^	Tobacco addiction	2/2	38/38	24 (11/13)	VAS score	ND	Non-acupuncture points	No penetration	No electrical stimulation	Sham EA type Q
Dias *et al*. 2014 Brazil^108^	Stress-related symptoms	13/8	33/20	5(3/2)	The scores of MSQ, PSQI and MBI-SS	T > C	Therapeutic acupoint	No penetration	No electrical stimulation	Sham EA type A
Lin *et al*. 2013Taiwan^109^	Insulin Resistance	2/2	16/15	1(1/0)	Plasma glucose	ND	Therapeutic acupoint	No penetration	No electrical stimulation	Sham EA type A
Michalek-Sauberer *et al*. 2007 Austria^110^	Perioperative pain	3/3	76/36	24(16/8)	5-point verbal rating scale, Time and amount of analgesic intake	ND	Therapeutic acupoint	No penetration	No electrical stimulation	Sham EA type A
Carlsson and Sjolund 2001 Sweden^111^	Chronic Low Back Pain	4/2	16/16	0(0/0)	VAS scores, Intake of analgesics, Sleep quality and level of activity	T > C?	Non-acupuncture points	No penetration	No electrical stimulation	Sham EA type Q

T, treatment group/real EA group; C, control group/sham EA group; NS, not stated; T > C, EA treatment group was significantly superior to sham EA control group; ND, no difference between EA and sham EA group; T < C, real EA group was significantly inferior to sham EA group; T > C?, the efficacy result of trial was reported as “T > C” without conducting the between-group analysis and with the original data not available; NRS, Numerical Rating Scale; SF_MPQ, Short-Form McGill Scale; FMRI, Functional magnetic resonance imaging; MASS, the Massachusetts General Hospital Acupuncture Sensation Scale; WOMAC, The Western Ontario and McMaster University Osteoarthritis Index; EPDS, Edinburgh Postnatal Depression Scale; HADS, Hospital Anxiety and Depression Scale; HDRS_17_, 17-item Hamilton Rating Scale for Depression; CGI, Clinical Global Impression; FMA, Fugl-Meyer Assessment; VAS, Visual Analog Scale; NIHSS, the National Institutes of Health Stroke Scale. BW, body weight; BMI, body mass index; BFM, body fat mass; BPI, Brief Pain Inventory; EDSS, Expanded Disability Status Scale; FAMS, Functional Assessment of multiple Sclerosis; C-MMASS, Modified Massachusetts General Hospital Acupuncture Sensation Scale – Chinese version; HR, Heart rate; MAP, mean arterial blood pressure; OLM, opioid-like medication; TNF-α, tumor necrosis factor-α; IL-8, interleukin-8; IL-10, interleukin-10; HDRS, Hamilton Depression Rating Scale; HAMD-17, the 17-item Hamilton Rating Scale for Depression; SDS, the Chinese-version Self-rating Depression Scale; SPT, single pain threshold; TST, temporal summation thresholds; R1, angle of muscle reaction; R2, passive range of motion; R2–R1, dynamic component; ISI, The Insomnia Severity Index; PSQI, the Pittsburgh Sleep Quality Index; CGI-S, the Clinical Global Impression - Severity scale; BPI-SF, Brief Pain Inventory Short Form; FACT-G instrument, the Functional Assessment of Cancer Therapy-General instrument; 24-item HAMD, the 24-item Hamilton Depression Rating Scale; TFM, Trunk Fat Mass; WC, Waist Circumference; HC, Hip Circumference; VAS-100, a 100-mm visual analogue scale; BOLD fMRI, blood oxygen level dependent functional magnetic resonance imaging; IPSS, the International Prostate Symptom Score; GCSI, the Gastroparesis Cardinal Symptom Index; SEPs, somatosensory evoked potentials; MVC, maximal voluntary contraction; NIH-CPSI, NIH-Chronic Prostatitis Symptom Index; NPQ, the Northwick Park Neck Pain Questionnaire; NHP, the Nottingham Health Profile; MSQ, Mini-Sleep Questionnaire; MBI-SS, the Maslach Burnout Inventory—Student Survey.

Note: Sham EA type A: sham EA on therapeutic acupoints plus no penetration plus no electrical stimulation; Sham EA type B: sham EA on therapeutic acupoints plus no penetration plus electrical stimulation; Sham EA type C: sham EA on therapeutic acupoints plus the same depth plus no electrical stimulation; Sham EA type D: sham EA on therapeutic acupoints plus superficial penetration plus no electrical stimulation; Sham EA type F: sham EA on nonspecific acupuncture points plus the same depth plus electrical stimulation; Sham EA type L: sham EA on non-acupuncture points plus the same depth plus electrical stimulation; Sham EA type M: sham EA on non-acupuncture points plus the same depth plus no electrical stimulation; Sham EA type N: sham EA on non-acupuncture points plus superficial penetration plus electrical stimulation; Sham EA type O: sham EA on non-acupuncture points plus superficial penetration plus no electrical stimulation; Sham EA type Q: sham EA on non-acupuncture points plus no penetration plus no electrical stimulation.

**Table 2:** Risk of bias of included studies.

Reference (author, year and country)	A	B	C	D	E	F	G	H
Ntritsou *et al*. 2014 USA^18^	+	+	+	−	+	+	?	+
Chu *et al*. 2012 Hong Kong^19^	+	+	+	−	+	+	?	+
Wang *et al*. 2010 Denmark^20^	+	+	+	−	+	+	?	+
Zyloney *et al*. 2010 USA^21^	+	−	+	−	+	?	?	+
Jubb *et al*. 2008 UK^22^	+	−	+	−	+	+	?	+
Chung *et al*. 2012 Hong Kong^23^	+	+	+	−	+	−	?	+
Barlas *et al*. 2006 UK^24^	+	−	+	−	+	?	?	+
Wayne *et al*. 2005 US^25^	+	+	+	−	+	+	?	+
Sahin *et al*. 2010 Turkey^26^	+	+	+	−	+	+	?	+
Fanti *et al*. 2003 Italy^27^	+	−	+	−	+	+	?	+
Hsing *et al*. 2012 Brazil^28^	+	−	+	−	+	+	?	+
Gosman-Hedström *et al*. 1998 Sweden^29^	+	+	+	−	+	+	?	+
Jing *et al*. 2009 China^30^	+	+	+	−	+	+	?	+
Kong *et al*. 2009 USA^31^	+	−	+	−	+	?	?	+
Darbandi *et al*. 2013 Iran^32^	+	?	+	−	+	?	?	+
Yang *et al*. 2012 China^33^	+	?	+	−	+	+	?	+
Liu *et al*. 2013 China^34^	+	+	+	−	+	+	?	+
Zhang *et al*. 2014 China^35^	+	+	+	−	+	+	?	+
Mao *et al*.2014 USA^36^	+	+	+	−	+	+	+	+
Leung *et al*. 2011 Hong Kong^37^	+	+	+	−	+	+	?	+
Rusy *et al*. 2002 USA^38^	+	?	+	−	+	+	?	+
Yang *et al*. 2014 China^39^	+	?	+	−	+	+	+	+
Chen *et al*. 2013 China^40^	+	?	+	−	+	+	?	+
Quispe-Cabanillas *et al*. 2012 Brazil^41^	+	?	+	−	+	+	+	+
Aranha *et al*. 2015 Brazil^42^	+	?	+	−	+	−	+	+
Yu *et al*. 2013 Hong Kong^43^	+	+	+	?	+	+	?	+
Li *et al*. 2010 China^44^	+	+	+	−	+	+	+	+
Zheng *et al*. 2007 Australia^45^	+	+	+	−	+	−	?	+
Li *et al*. 2013 China^46^	+	?	+	?	+	+	?	+
Lin *et al*. 2002 Taiwan^47^	+	?	+	?	+	+	?	+
Chen *et al*. 2013 Taiwan^48^	+	?	+	−	+	−	?	+
Schukro *et al*. 2014 Austria^49^	+	?	+	?	+	+	?	+
Yu *et al*. 2014 China^50^	+	?	+	−	+	+	?	+
Xie *et al*. 2014 China^51^	+	?	+	?	+	+	?	+
Sim *et al*. 2002 Singapore^52^	+	?	+	−	+	+	?	+
Song *et al*. 2009 China^53^	+	?	+	?	+	?	?	+
Shafshak 1995 Egypt^54^	+	?	+	?	?	?	?	+
Franasiak *et al*. 2012 USA^55^	+	+	+	−	+	+	?	+
Naslund *et al*. 2002 Sweden^56^	+	?	+	−	+	?	?	+
Shen *et al*. 2000 US^57^	+	+	+	−	+	+	?	−
Wyon *et al*. 2004 Sweden^58^	+	+	+	−	+	+	?	−
Zhang *et al*. 2012 Hong Kong^59^	+	?	+	−	+	+	+	−
Zheng *et al*. 2010 Australia^60^	+	+	+	−	+	+	?	+
Ma *et al*. 2010 China^61^	+	+	+	−	+	+	?	+
Wang *et al*. 2014 Taiwan^62^	+	?	+	−	+	+	?	+
Yeung *et al*. 2011 Hong Kong^63^	+	+	+	−	+	+	+	+
Chan *et al*. 2014 Taiwan^64^	+	+	+	−	+	+	+	+
Man *et al*. 2014 Hong Kong^65^	+	+	+	−	+	+	−	+
Oh *et al*. 2013 Australia^66^	+	+	−	−	−	+	?	+
Wong *et al*. 2006 Hong Kong^67^	+	+	+	−	+	+	?	+
Song *et al*. 2007 USA^68^	+	?	+	?	+	?	?	+
Cameron *et al*. 2011 Australia^69^	+	+	+	−	+	−	?	−
Darbandi *et al*. 2014 Iran^70^	+	?	+	−	+	+	?	+
Wei *et al*. 2008 China^71^	+	+	+	?	?	+	?	?
Wang *et al*. 2007 USA^72^	+	+	+	−	+	+	?	+
Kvorning *et al*. 2003 Sweden^73^	+	?	+	−	+	+	?	+
Kvorning *et al*. 2003 Sweden^74^	+	?	+	−	+	+	?	+
Yang *et al*.2010 China^75^	+	?	+	−	+	+	+	+
Sahmeddini *et al*.2010 Iran^76^	+	+	+	?	+	+	?	+
Wang *et al*. 2007 China^77^	+	?	+	−	+	+	?	?
Jia *et al*. 2009 China^78^	+	?	+	?	+	+	?	−
Wang et al. 2013 China^79^	+	+	+	−	+	+	+	+
Andreescu *et al*. 2011 Canada^80^	+	?	+	−	+	+	+	+
Yeung *et al*. 2009 Hong Kong^81^	+	+	+	−	+	+	?	−
Chen *et al*. 2014 Taiwan^82^	+	?	+	?	+	+	?	+
Wang *et al*. 2008 China^83^	+	?	+	−	+	+	?	+
Meissner *et al*. 2004 Germany^84^	+	+	+	−	+	+	?	+
Zhou *et al*. 2012 China^85^	+	?	+	−	+	+	?	+
Yeh *et al*. 2012 Taiwan^86^	+	+	+	−	+	+	+	+
Yu *et al*. 2011 Taiwan^87^	+	?	+	−	+	+	?	+
Ma *et al*. 2011 China^88^	+	+	+	−	+	+	?	+
Li *et al*. 2013 China^89^	+	?	+	?	+	+	?	+
Deluze *et al*. 1992 Switzerland^90^	+	+	+	−	+	+	?	−
Ng *et al*. 2013 Hong Kong^91^	+	+	+	−	+	+	+	+
Lee and Lee 2009 Republic of Korea^92^	+	?	+	−	+	+	?	+
Tam *et al*. 2007 Hong Kong^93^	+	+	+	−	+	−	+	+
Kvorning and Akeson 2010 Sweden^94^	+	?	+	−	+	+	?	+
Waite and Clough 1998 UK^95^	+	?	+	?	+	+	?	+
Holzer *et al*. 2011 Austria^96^	+	?	+	−	+	+	?	+
Sator-Katzenschlager *et al*. 2006 Austria^97^	+	?	+	−	+	+	?	+
Liu *et al*. 2011 China^98^	+	+	+	−	+	+	+	−
Liu *et al*. 2014 China^99^	+	+	+	−	+	+	+	+
Maeda *et al*. 2013 USA^100^	+	?	+	−	+	+	?	?
Shi *et al*. 2011 China^101^	+	?	+	?	+	+	?	+
Dias *et al*. 2010 Brazil^102^	+	?	+	?	+	+	?	−
Zhang *et al*. 2013 Hong Kong^103^	+	?	+	?	+	+	?	+
Sangdee *et al*. 2002 Thailand^104^	+	?	+	−	+	+	?	+
Johansson *et al*. 2001 Sweden^105^	+	+	+	−	+	+	?	+
Hopwood *et al*. 2008 UK^106^	+	+	+	−	+	−	?	−
White *et al*. 1998 England^107^	+	+	+	−	+	−	?	−
Dias *et al*. 2014 Brazil^108^	+	?	+	−	+	+	?	−
Lin *et al*. 2013Taiwan^109^	+	+	+	−	+	+	?	+
Michalek-Sauberer *et al*. 2007 Austria^110^	+	+	+	−	+	+	?	−
Carlsson and Sjolund 2001 Sweden^111^	+	+	+	−	+	+	?	+

Note: A, Adequate sequence generation; B, Allocation Concealment; C, Blinding (participants); D, Blinding (personnel); E, Blinding (outcome assessor); F, Incomplete outcome data addressed; G, Free of selective reporting; H, Free of other bias. +, Yes; −, No; ?, Unclear.

**Table 3:** Summary of effect result within different type of sham electro-acupuncture methods and electro-acupuncture indications.

electro-acupuncture (EA) indications	The type of sham EA method										The NO. of reference included
	Sham EA type A 26 control groups	Sham EA type B 7 control groups	Sham EA type C 7 control groups	Sham EA type D 6 control groups	Sham EA type F 7 control groups	Sham EA type L 17 control groups	Sham EA type M 3 control groups	Sham EA type N 4 control groups	Sham EA type O 14 control groups	Sham EA type Q 10 control groups	
Pain 32 RCTs	T > C 8 comparisons ND 7 comparisons	T > C 3 comparisons ND 1 comparison	T > C 1 comparison ND 3 comparisons	T > C 1 comparison		ND 2 comparisons	T > C 1 comparison	T > C 1 comparison	T > C 1 comparison ND 2 comparisons	ND 2 comparisons T > C? 1 comparison	T > C 16 ND 17 T > C? 1
Obesity 4 RCTs			T > C 1 comparison			T > C 2 comparisons			T > C 1 comparison ND 1 comparison		T > C 4 ND 1
Anesthesia 8 RCTs	T > C 2 comparisons ND 1 comparison T < C 1 comparison					ND 2 comparisons T > C? 1 comparison			T > C 1 comparison		T > C 3 ND 3 T < C 1 T > C? 1
Stroke 7 RCTs	ND 1 comparison	ND 1 comparison	T > C 1 comparison	T > C 1 comparison ND 1 comparison						T > C 1 comparison ND 1 comparison	T > C 3 ND 4
Depression 6 RCTs		T > C 2 comparisons ND 1 comparison				T > C 1 comparison	ND 1 comparison		ND 1 comparison		T > C 3 ND 3
Primary dysmenorrhea and (or) Menstrual Pain 4 RCTs					T > C 2 comparisons ND 2 comparisons	T > C 2 comparisons ND 2 comparisons					T > C 4 ND 4
Substance abuse 3 RCTs				T > C 1 comparison				T > C 1 comparison		ND 1 comparison	T > C 2 ND 1
Healthy 3 RCTs				T > C 1 comparison		T > C 1 comparison ND 2 comparisons					T > C 2 ND 2
Osteoarthritis 2 RCTs	T > C 2 comparisons										T > C 2
Migraine 2 RCTs					T > C 1 comparison	ND 1 comparison					T > C 1 ND 1
Nausea and Vomiting 2 RCTs					T > C 1 comparison				T > C 1 comparison	T > C 1 comparison	T > C 3
Postoperative ileus 2 RCTs									T > C 2 comparisons		T > C 2
Insomnia 2 RCTs	ND 1 comparison							ND 1 comparison		T > C 1 comparison	T > C 1 ND 2
benign prostate hyperplasia 2 RCTs						T > C 1 comparison			T > C 1 comparison		T > C 2
Diabetic mellitus 2 RCTs	ND 1 comparison							T > C 1 comparison			T > C 1 ND 1
Carpal tunnel syndrome 1 RCTs										T > C 2 comparisons	T > C 2
Rheumatoid arthritis 1 RCTs				ND 1 comparison							ND 1
Whiplash-associated disorders 1 RCTs							T > C 1 comparison				T > C 1
Constipation 1 RCTs						T > C 1 comparison					T > C 1
Multiple sclerosis 1 RCTs									T > C 1 comparison		T > C 1
Tinnitus 1 RCTs	ND 1 comparison										ND 1
Auditory hallucination 1 RCTs									T > C 1 comparison		T > C 1
ADHD (Attention deficit hyperactivity disorder) 1 RCTs			T > C 1 comparison								T > C 1
PCOS (Polycystic Ovary Syndrome) 1 RCTs										ND 1 comparison	ND 1
hot flushes in postmenopausal women 1 RCTs									ND 1 comparison		ND 1
Postpartum Insufficient Lactation 1 RCTs					T > C 1 comparison						T > C 1
Cardiac ischemia-reperfusion injury 1 RCTs			T > C 1 comparison								T > C 1
Stress-related symptoms 1 RCTs	T > C 1 comparison										T > C 1
The positive rate of efficacy result	T > C 13 ND 12 T < C 1 T > C? 0 53.8% (14/26 comparisons)	T > C 5 ND 3 62.5% (5/8 comparisons)	T > C 5 ND 3 62.5% (5/8 comparisons)	T > C 4 ND 2 66.7% (4/6 comparisons)	T > C 5 ND 2 71.4% (5/7 comparisons)	T > C 8 ND 9 T > C? 1 44.4% (8/18 comparisons)	T > C 2 ND 1 66.7% (2/3 comparisons)	T > C 3 ND 1 75% (3/4 comparisons)	T > C 9 ND 5 64.3% (9/14 comparisons)	T > C 5 ND 5 T > C? 1 45.5% (5/11 comparisons)	T > C 59 ND 43 T < C 1 T > C? 2 57.1% (60/105 comparisons)

NOTE: T > C, EA treatment group was significantly superior to sham EA control group; ND, no difference between EA and sham EA group; T < C, real EA group was significantly inferior to sham EA group; T > C?, the efficacy result of trial was reported as “T > C” without conducting the between-group analysis and with the original data not available.

## References

[b1] Medical Research Council (1948). Streptomycin Treatment of Pulmonary Tuberculosis. Br. Med. J. 2, 769–782 (1948). 18890300 PMC2091872

[b2] Meldrum M. L. A brief history of the randomized controlled trial. From oranges and lemons to the gold standard. Hematol. Oncol. Clin. North. Am. 14, 745–760 (2000). 10949771 10.1016/s0889-8588(05)70309-9

[b3] Guidance for Industry Statistical Principles for Clinical Trials ICH Topic E9. (2003). Available at: http://www.hc-sc.gc.ca/dhp-mps/prodpharma/applic-demande/guide-ld/ich/efficac/e9-eng.php#fnb1-ref. (Accessed: 6th November 2015).

[b4] Boutron I. . Reporting methods of blinding in randomized trials assessing nonpharmacological treatments. PLoS Med. 4, e61 (2007). 17311468 10.1371/journal.pmed.0040061 PMC1800311

[b5] Gaw A. C., Chang L. W. & Shaw L. C. Efficacy of acupuncture on osteoarthritic pain. A controlled, double-blind study. N. Engl. J. Med. 293, 375–378 (1975). 1097921 10.1056/NEJM197508212930803

[b6] Hammerschlag R. . Randomized Controlled Trials of Acupuncture (1997–2007): An Assessment of Reporting Quality with a CONSORT- and STRICTA-Based Instrument. Evid. Based Complement. Alternat. Med. 2011, 183910 (2011). 20953418 10.1155/2011/183910 PMC2952291

[b7] Langevin H. M. . Paradoxes in acupuncture research: strategies for moving forward. Evid. Based Complement. Alternat. Med. 2011, 180805 (2011). 20976074 10.1155/2011/180805 PMC2957136

[b8] Dincer F. & Linde K. Sham interventions in randomized clinical trials of acupuncture–a review. Complement. Ther. Med. 11, 235–242 (2003). 15022656 10.1016/s0965-2299(03)00124-9

[b9] He W. . Review of controlled clinical trials on acupuncture versus sham acupuncture in Germany. J. Tradit. Chin. Med. 33, 403–407 (2013). 24024341 10.1016/s0254-6272(13)60187-9

[b10] Zhang C. S., Yang A. W., Zhang A. L., May B. H. & Xue C. C. Sham control methods used in ear-acupuncture/ear-acupressure randomized controlled trials: a systematic review. J. Altern. Complement. Med. 20, 147–161 (2014). 24138333 10.1089/acm.2013.0238 PMC3948482

[b11] Liu A. J. . Electroacupuncture for Acute Ischemic Stroke: A Meta-Analysis of Randomized Controlled Trials. Am. J. Chin. Med. 43, 1541–1566 (2015). 26621442 10.1142/S0192415X15500883

[b12] Wang W. W., Xie C. L., Lu L. & Zheng G. Q. A systematic review and meta-analysis of Baihui (GV20)-based scalp acupuncture in experimental ischemic stroke. Sci. Rep. 4, 3981 (2014). 24496233 10.1038/srep03981 PMC5379241

[b13] Mayor D. An exploratory review of the electroacupuncture literature: clinical applications and endorphin mechanisms. Acupunct. Med. 31, 409–415 (2013). 23917395 10.1136/acupmed-2013-010324

[b14] Fukazawa Y., Maeda T. & Kishioka S. The pharmacological mechanisms of electroacupuncture. Curr. Opin. Investig. Drugs. 10, 62–69 (2009). 19127488

[b15] Kreiner M., Zaffaroni A., Alvarez R. & Clark G. Validation of a simplified sham acupuncture technique for its use in clinical research: a randomised, single blind, crossover study. Acupunct. Med. 28, 33–36 (2010). 20351375 10.1136/aim.2009.001735

[b16] Tough E. A., White A. R., Richards S. H., Lord B. & Campbell J. L. Developing and validating a sham acupuncture needle. Acupunct. Med. 27, 118–122 (2009). 19734382 10.1136/aim.2009.000737

[b17] Lee H. . Non-penetrating sham needle, is it an adequate sham control in acupuncture research? Complement. Ther. Med. 19 Suppl 1, S41–48 (2011). 21195294 10.1016/j.ctim.2010.12.002

[b18] Ntritsou V. . Effect of perioperative electroacupuncture as an adjunctive therapy on postoperative analgesia with tramadol and ketamine in prostatectomy: a randomised sham-controlled single-blind trial. Acupunct. Med. 32, 215–222 (2014). 24480836 10.1136/acupmed-2013-010498

[b19] Chu W. C. . Does acupuncture therapy alter activation of neural pathway for pain perception in irritable bowel syndrome?: a comparative study of true and sham acupuncture using functional magnetic resonance imaging. J. Neurogastroenterol. Motil. 18, 305–316 (2012). 22837879 10.5056/jnm.2012.18.3.305 PMC3400819

[b20] Wang K., Bugge J. & Bugge S. A randomised, placebo-controlled trial of manual and electrical acupuncture for the treatment of tinnitus. Complement. Ther. Med. 18, 249–255 (2010). 21130361 10.1016/j.ctim.2010.09.005

[b21] Zyloney C. E. . Imaging the functional connectivity of the Periaqueductal Gray during genuine and sham electroacupuncture treatment. Mol. Pain. 6, 80 (2010). 21080967 10.1186/1744-8069-6-80 PMC2993660

[b22] Jubb R. W. . A blinded randomised trial of acupuncture (manual and electroacupuncture) compared with a non-penetrating sham for the symptoms of osteoarthritis of the knee. Acupunct. Med. 26, 69–78 (2008). 18591906 10.1136/aim.26.2.69

[b23] Chung K. F. . Randomized non-invasive sham-controlled pilot trial of electroacupuncture for postpartum depression. J. Affect. Disord. 142, 115–121 (2012). 22840621 10.1016/j.jad.2012.04.008

[b24] Barlas P., Ting S. L., Chesterton L. S., Jones P. W. & Sim J. Effects of intensity of electroacupuncture upon experimental pain in healthy human volunteers: a randomized, double-blind, placebo-controlled study. Pain 122, 81–89 (2006). 16527396 10.1016/j.pain.2006.01.012

[b25] Wayne P. M. . Acupuncture for upper-extremity rehabilitation in chronic stroke: a randomized sham-controlled study. Arch. Phys. Med. Rehabil. 86, 2248–2255 (2005). 16344019 10.1016/j.apmr.2005.07.287

[b26] Sahin N., Ozcan E., Sezen K., Karatas O. & Issever H. Efficacy of acupunture in patients with chronic neck pain–a randomised, sham controlled trial. Acupunct. Electrother. Res. 35, 17–27 (2010). 20578644 10.3727/036012910803860959

[b27] Fanti L. . Electroacupuncture analgesia for colonoscopy. a prospective, randomized, placebo-controlled study. Am. J. Gastroenterol. 98, 312–316 (2003). 12591047 10.1111/j.1572-0241.2003.07231.x

[b28] Hsing W. T., Imamura M., Weaver K., Fregni F. & Azevedo Neto R. S. Clinical effects of scalp electrical acupuncture in stroke: a sham-controlled randomized clinical trial. J. Altern. Complement. Med. 18, 341–346 (2012). 22489807 10.1089/acm.2011.0131

[b29] Gosman-Hedstrom G. . Effects of acupuncture treatment on daily life activities and quality of life: a controlled, prospective, and randomized study of acute stroke patients. Stroke 29, 2100–2108 (1998). 9756589 10.1161/01.str.29.10.2100

[b30] Jing C. . Electro-acupuncture versus sham electro-acupuncture for auditory hallucinations in patients with schizophrenia: a randomized controlled trial. Clin. Rehabil. 23, 579–588 (2009). 19470551 10.1177/0269215508096172

[b31] Kong J. . An fMRI study on the interaction and dissociation between expectation of pain relief and acupuncture treatment. NeuroImage 47, 1066–1076 (2009). 19501656 10.1016/j.neuroimage.2009.05.087 PMC2742363

[b32] Darbandi S. . Effects of body electroacupuncture on plasma leptin concentrations in obese and overweight people in Iran: a randomized controlled trial. Altern. Ther. Health Med. 19, 24–31 (2013). 23594450

[b33] Yang C., An L., Han R., Kang X. & Wang B. Effects of combining electroacupuncture with general anesthesia induced by sevoflurane in patients undergoing supratentorial craniotomy and improvements in their clinical recovery profile & blood enkephalin. Acupunct. Electrother. Res. 37, 125–138 (2012). 23156204 10.3727/036012912x13831831256249

[b34] Liu Y., Liu L. & Wang X. Electroacupuncture at points Baliao and Huiyang (BL35) for post-stroke detrusor overactivity. Neural. Regen. Res. 8, 1663–1672 (2013). 25206463 10.3969/j.issn.1673-5374.2013.18.004 PMC4145909

[b35] Zhang Z. . Electroacupuncture at ST36 accelerates the recovery of gastrointestinal motility after colorectal surgery: a randomised controlled trial. Acupunct. Med. 32, 223–226 (2014). 24739815 10.1136/acupmed-2013-010490

[b36] Mao J. J. . Electroacupuncture for fatigue, sleep, and psychological distress in breast cancer patients with aromatase inhibitor-related arthralgia: a randomized trial. Cancer 120, 3744–3751 (2014). 25077452 10.1002/cncr.28917 PMC4239308

[b37] Leung W. W., Jones A. Y., Ng S. S., Wong C. Y. & Lee J. F. Electroacupuncture in reduction of discomfort associated with barostat-induced rectal distension–a randomized controlled study. J. Gastrointest. Surg. 15, 660–666 (2011). 21327534 10.1007/s11605-011-1446-5

[b38] Rusy L. M., Hoffman G. M. & Weisman S. J. Electroacupuncture prophylaxis of postoperative nausea and vomiting following pediatric tonsillectomy with or without adenoidectomy. Anesthesiology 96, 300–305 (2002). 11818760 10.1097/00000542-200202000-00013

[b39] Yang M. . Electroacupuncture stimulation at sub-specific acupoint and non-acupoint induced distinct brain glucose metabolism change in migraineurs: a PET-CT study. J. Transl. Med. 12, 351 (2014). 25496446 10.1186/s12967-014-0351-6 PMC4279794

[b40] Chen H. . Electroacupuncture treatment for pancreatic cancer pain: a randomized controlled trial. Pancreatology 13, 594–597 (2013). 24280575 10.1016/j.pan.2013.10.007

[b41] Quispe-Cabanillas J. G. . Impact of electroacupuncture on quality of life for patients with Relapsing-Remitting Multiple Sclerosis under treatment with immunomodulators: a randomized study. BMC Complement. Altern. Med. 12, 209 (2012). 23126260 10.1186/1472-6882-12-209 PMC3565890

[b42] Aranha M. F., Muller C. E. & Gaviao M. B. Pain intensity and cervical range of motion in women with myofascial pain treated with acupuncture and electroacupuncture: a double-blinded, randomized clinical trial. Braz. J. Phys. Ther. 19, 34–43 (2015). 25714602 10.1590/bjpt-rbf.2014.0066 PMC4351606

[b43] Yu D. T. & Jones A. Y. Physiological changes associated with de qi during electroacupuncture to LI4 and LI11: a randomised, placebo-controlled trial. Acupunct. Med. 31, 143–150 (2013). 23542157 10.1136/acupmed-2012-010280

[b44] Li S. . Randomized-controlled study of treating attention deficit hyperactivity disorder of preschool children with combined electro-acupuncture and behavior therapy. Complement. Ther. Med. 18, 175–183 (2010). 21056840 10.1016/j.ctim.2010.08.002

[b45] Zheng Z. . The effect of electroacupuncture on opioid-like medication consumption by chronic pain patients: a pilot randomized controlled clinical trial. Eur. J. Pain 12, 671–676 (2008). 18035566 10.1016/j.ejpain.2007.10.003

[b46] Li G., Li S., Wang B. & An L. The effect of electroacupuncture on postoperative immunoinflammatory response in patients undergoing supratentorial craniotomy. Exp. Ther. Med. 6, 699–702 (2013). 24137250 10.3892/etm.2013.1225 PMC3786813

[b47] Lin J. G. . The effect of high and low frequency electroacupuncture in pain after lower abdominal surgery. Pain 99, 509–514 (2002). 12406527 10.1016/S0304-3959(02)00261-0

[b48] Chen C. Y. . The influence of electro-acupuncture stimulation to female constipation patients. Am. J. Chin. Med. 41, 301–313 (2013). 23548121 10.1142/S0192415X13500225

[b49] Schukro R. P., Heiserer C., Michalek-Sauberer A., Gleiss A. & Sator-Katzenschlager S. The effects of auricular electroacupuncture on obesity in female patients–a prospective randomized placebo-controlled pilot study. Complement. Ther. Med. 22, 21–25 (2014). 24559812 10.1016/j.ctim.2013.10.002

[b50] Yu J. B. . Effect of electroacupuncture at Zusanli (ST36) and Sanyinjiao (SP6) acupoints on adrenocortical function in etomidate anesthesia patients. Med. Sci. Monit. 20, 406–412 (2014). 24621826 10.12659/MSM.890111 PMC3958570

[b51] Xie Y. H., Chai X. Q., Wang Y. L., Gao Y. C. & Ma J. Effect of electro-acupuncture stimulation of Ximen (PC4) and Neiguan (PC6) on remifentanil-induced breakthrough pain following thoracal esophagectomy. J. Huazhong Univ. Sci. Technolog. Med. Sci. 34, 569–574 (2014). 25135729 10.1007/s11596-014-1317-x

[b52] Sim C. K., Xu P. C., Pua H. L., Zhang G. & Lee T. L. Effects of electroacupuncture on intraoperative and postoperative analgesic requirement. Acupunct. Med. 20, 56–65 (2002). 12216602 10.1136/aim.20.2-3.56

[b53] Song C., Halbreich U., Han C., Leonard B. E. & Luo H. Imbalance between pro- and anti-inflammatory cytokines, and between Th1 and Th2 cytokines in depressed patients: the effect of electroacupuncture or fluoxetine treatment. Pharmacopsychiatry 42, 182–188 (2009). 19724980 10.1055/s-0029-1202263

[b54] Shafshak T. S. Electroacupuncture and exercise in body weight reduction and their application in rehabilitating patients with knee osteoarthritis. Am. J. Chin. Med. 23, 15–25 (1995). 7598088 10.1142/S0192415X95000043

[b55] Pastore L. M., Williams C. D., Jenkins J. & Patrie J. T. True and sham acupuncture produced similar frequency of ovulation and improved LH to FSH ratios in women with polycystic ovary syndrome. J. Clin. Endocrinol. Metab. 96, 3143–3150 (2011). 21816787 10.1210/jc.2011-1126 PMC3200239

[b56] Naslund J., Naslund U. B., Odenbring S. & Lundeberg T. Sensory stimulation (acupuncture) for the treatment of idiopathic anterior knee pain. J. Rehabil. Med. 34, 231–238 (2002). 12392239 10.1080/165019702760279233

[b57] Shen J. . Electroacupuncture for control of myeloablative chemotherapy-induced emesis: A randomized controlled trial. J. A. M. A. 284, 2755–2761 (2000). 10.1001/jama.284.21.2755 11105182

[b58] Wyon Y., Wijma K., Nedstrand E. & Hammar M. A comparison of acupuncture and oral estradiol treatment of vasomotor symptoms in postmenopausal women. Climacteric 7, 153–164 (2004). 15497904 10.1080/13697130410001713814

[b59] Zhang Z. J. . Dense cranial electroacupuncture stimulation for major depressive disorder–a single-blind, randomized, controlled study. PloS One 7, e29651 (2012). 22238631 10.1371/journal.pone.0029651 PMC3253099

[b60] Zheng Z. . Acupuncture analgesia for temporal summation of experimental pain: a randomised controlled study. Eur. J. Pain 14, 725–731 (2010). 20045360 10.1016/j.ejpain.2009.11.006

[b61] Ma Y. X. . A comparative study on the immediate effects of electroacupuncture at Sanyinjiao (SP6), Xuanzhong (GB39) and a non-meridian point, on menstrual pain and uterine arterial blood flow, in primary dysmenorrhea patients. Pain Med. 11, 1564–1575 (2010). 21199306 10.1111/j.1526-4637.2010.00949.x

[b62] Wang B. H. . Selection of acupoints for managing upper-extremity spasticity in chronic stroke patients. Clin. Interv. Aging 9, 147–156 (2014). 24453485 10.2147/CIA.S53814 PMC3894143

[b63] Yeung W. F. . Electroacupuncture for residual insomnia associated with major depressive disorder: a randomized controlled trial. Sleep 34, 807–815 (2011). 21629370 10.5665/SLEEP.1056 PMC3099500

[b64] Chan Y. Y. . Clinical efficacy of acupuncture as an adjunct to methadone treatment services for heroin addicts: a randomized controlled trial. Am. J. Chin. Med. 42, 569–586 (2014). 24871652 10.1142/S0192415X14500372

[b65] Man S. C. . A pilot controlled trial of a combination of dense cranial electroacupuncture stimulation and body acupuncture for post-stroke depression. BMC Complement. Altern. Med. 14, 255 (2014). 25038733 10.1186/1472-6882-14-255 PMC4223407

[b66] Oh B. . Acupuncture for treatment of arthralgia secondary to aromatase inhibitor therapy in women with early breast cancer: pilot study. Acupunct. Med. 31, 264–271 (2013). 23722951 10.1136/acupmed-2012-010309

[b67] Wong R. H. . Analgesic effect of electroacupuncture in postthoracotomy pain: a prospective randomized trial. Ann. Thorac. Surg. 81, 2031–2036 (2006). 16731125 10.1016/j.athoracsur.2005.12.064

[b68] Song Y., Zhou D., Fan J., Luo H. & Halbreich U. Effects of electroacupuncture and fluoxetine on the density of GTP-binding-proteins in platelet membrane in patients with major depressive disorder. J. Affect. Disord. 98, 253–257 (2007). 16919758 10.1016/j.jad.2006.07.012

[b69] Cameron I. D., Wang E. & Sindhusake D. A randomized trial comparing acupuncture and simulated acupuncture for subacute and chronic whiplash. Spine 36, E1659–1665 (2011). 21494196 10.1097/BRS.0b013e31821bf674

[b70] Darbandi M. . Auricular or body acupuncture: which one is more effective in reducing abdominal fat mass in Iranian men with obesity: a randomized clinical trial. J. Diabetes Metab. Disord. 13, 92 (2014). 25505744 10.1186/s40200-014-0092-3 PMC4261582

[b71] Wei L., Wang H., Han Y. & Li C. Clinical observation on the effects of electroacupuncture at Shaoze (SI 1) in 46 cases of postpartum insufficient lactation. J. Tradit. Chin. Med. 28, 168–172 (2008). 19004195 10.1016/s0254-6272(08)60038-2

[b72] Wang S. M., Punjala M., Weiss D., Anderson K. & Kain Z. N. Acupuncture as an adjunct for sedation during lithotripsy. J. Altern. Complement. Med. 13, 241–246 (2007). 17388767 10.1089/acm.2006.6262

[b73] Kvorning N., Christiansson C. & Akeson J. Acupuncture facilitates neuromuscular and oculomotor responses to skin incision with no influence on auditory evoked potentials under sevoflurane anaesthesia. Acta Anaesthesiol. Scand. 47, 1073–1078 (2003). 12969098 10.1034/j.1399-6576.2003.00224.x

[b74] Kvorning N., Christiansson C., Beskow A., Bratt O. & Akeson J. Acupuncture fails to reduce but increases anaesthetic gas required to prevent movement in response to surgical incision. Acta Anaesthesiol. Scand. 47, 818–822 (2003). 12859301 10.1034/j.1399-6576.2003.00171.x

[b75] Yang L. . Cardioprotective effects of electroacupuncture pretreatment on patients undergoing heart valve replacement surgery: a randomized controlled trial. Ann. Thorac. Surg. 89, 781–786 (2010). 20172127 10.1016/j.athoracsur.2009.12.003

[b76] Sahmeddini M. A., Farbood A. & Ghafaripuor S. Electro-acupuncture for pain relief after nasal septoplasty: a randomized controlled study. J. Altern. Complement. Med. 16, 53–57 (2010). 20001536 10.1089/acm.2009.0288

[b77] Wang W. . Study on the regulatory effect of electro-acupuncture on hegu point (LI4) in cerebral response with functional magnetic resonance imaging. Chin. J. Integr. Med. 13, 10–16 (2007). 17578311 10.1007/s11655-007-0010-3

[b78] Jia C. S. . Electroacupuncture at Qiuxu (GB 40) for treatment of migraine–a clinical multicentral random controlled study. J. Tradit. Chin. Med. 29, 43–49 (2009). 19514188 10.1016/s0254-6272(09)60030-3

[b79] Wang Y. . Electroacupuncture for moderate and severe benign prostatic hyperplasia: a randomized controlled trial. PloS One 8, e59449 (2013). 23593139 10.1371/journal.pone.0059449 PMC3625218

[b80] Andreescu C., Glick R. M., Emeremni C. A., Houck P. R. & Mulsant B. H. Acupuncture for the treatment of major depressive disorder: a randomized controlled trial. J. Clin. Psychiatry 72, 1129–1135 (2011). 21672495 10.4088/JCP.10m06105 PMC10536993

[b81] Yeung W. F., Chung K. F., Zhang S. P., Yap T. G. & Law A. C. Electroacupuncture for primary insomnia: a randomized controlled trial. Sleep 32, 1039–1047 (2009). 19725255 10.1093/sleep/32.8.1039 PMC2717194

[b82] Chen W. T., Chang F. C., Chen Y. H. & Lin J. G. An Evaluation of Electroacupuncture at the Weizhong Acupoint (BL-40) as a Means of Relieving Pain Induced by Extracorporeal Shock Wave Lithotripsy. Evid. Based Complement. Alternat. Med. 2014, 592319 (2014). 25152761 10.1155/2014/592319 PMC4135135

[b83] Wang C. P., Kao C. H., Chen W. K., Lo W. Y. & Hsieh C. L. A single-blinded, randomized pilot study evaluating effects of electroacupuncture in diabetic patients with symptoms suggestive of gastroparesis. J. Altern. Complement. Med. 14, 833–839 (2008). 18721079 10.1089/acm.2008.0107

[b84] Meissner W. . Acupuncture decreases somatosensory evoked potential amplitudes to noxious stimuli in anesthetized volunteers. Anesth. Analg. 98, 141–147 (2004). 14693608 10.1213/01.ANE.0000096191.07929.44

[b85] Zhou S. . Bilateral effects of 6 weeks’ unilateral acupuncture and electroacupuncture on ankle dorsiflexors muscle strength: a pilot study. Arch. Phys. Med. Rehabil. 93, 50–55 (2012). 22075372 10.1016/j.apmr.2011.08.010

[b86] Yeh B. Y. . Effect of electroacupuncture in postanesthetic shivering during regional anesthesia: a randomized controlled trial. BMC Complement. Altern. Med. 12, 233 (2012). 23181618 10.1186/1472-6882-12-233 PMC3526538

[b87] Yu J. S., Shen K. H., Chen W. C., Her J. S. & Hsieh C. L. Effects of electroacupuncture on benign prostate hyperplasia patients with lower urinary tract symptoms: a single-blinded, randomized controlled trial. Evid. Based Complement. Alternat. Med. 2011, 303198 (2011). 21584227 10.1155/2011/303198 PMC3092516

[b88] Ma W. . Effects of Sanyinjiao (SP6) with electroacupuncture on labour pain in women during labour. Complement. Ther. Med. 19 Suppl 1, S13–18 (2011). 21195290 10.1016/j.ctim.2010.09.001

[b89] Li G., Li S., An L. & Wang B. Electroacupuncture alleviates intraoperative immunosuppression in patients undergoing supratentorial craniotomy. Acupunct. Med. 31, 51–56 (2013). 23315447 10.1136/acupmed-2012-010254

[b90] Deluze C., Bosia L., Zirbs A., Chantraine A. & Vischer T. L. Electroacupuncture in fibromyalgia: results of a controlled trial. B. M. J. 305, 1249–1252 (1992). 10.1136/bmj.305.6864.1249 PMC1883744 1477566

[b91] Ng S. S. . Electroacupuncture reduces duration of postoperative ileus after laparoscopic surgery for colorectal cancer. Gastroenterology 144, 307–313.e301 (2013). 10.1053/j.gastro.2012.10.050 23142625

[b92] Lee S. H. & Lee B. C. Electroacupuncture relieves pain in men with chronic prostatitis/chronic pelvic pain syndrome: three-arm randomized trial. Urology 73, 1036–1041 (2009). 19394499 10.1016/j.urology.2008.10.047

[b93] Tam L. S., Leung P. C., Li T. K., Zhang L. & Li E. K. Acupuncture in the treatment of rheumatoid arthritis: a double-blind controlled pilot study. BMC Complement. Altern. Med. 7, 35 (2007). 17980044 10.1186/1472-6882-7-35 PMC2174514

[b94] Kvorning N. & Akeson J. Plasma adrenaline increases in anesthetized patients given electro-acupuncture before surgery. Pain Med. 11, 1126–1131 (2010). 20545874 10.1111/j.1526-4637.2010.00878.x

[b95] Waite N. R. & Clough J. B. A single-blind, placebo-controlled trial of a simple acupuncture treatment in the cessation of smoking. Br. J. Gen. Pract. 48, 1487–1490 (1998). 10024707 PMC1313196

[b96] Holzer A. . Auricular acupuncture for postoperative pain after gynecological surgery: a randomized controlled trail. Minerva Anestesiol. 77, 298–304 (2011). 21441884

[b97] Sator-Katzenschlager S. M. . Auricular electro-acupuncture as an additional perioperative analgesic method during oocyte aspiration in IVF treatment. Hum. Reprod. 21, 2114–2120 (2006). 16679325 10.1093/humrep/del110

[b98] Liu C. Z. . Immediate analgesia effect of single point acupuncture in primary dysmenorrhea: a randomized controlled trial. Pain Med. 12, 300–307 (2011). 21166767 10.1111/j.1526-4637.2010.01017.x

[b99] Liu C. Z. . A randomized controlled trial of single point acupuncture in primary dysmenorrhea. Pain Med. 15, 910–920 (2014). 24636695 10.1111/pme.12392

[b100] Maeda Y. . Acupuncture-evoked response in somatosensory and prefrontal cortices predicts immediate pain reduction in carpal tunnel syndrome. Evid. Based Complement. Alternat. Med. 2013, 795906 (2013). 23843881 10.1155/2013/795906 PMC3703406

[b101] Shi G. X. . Effects of acupuncture at Sanyinjiao (SP6) on prostaglandin levels in primary dysmenorrhea patients. Clin. J. Pain 27, 258–261 (2011). 21358291 10.1097/AJP.0b013e3181fb27ae

[b102] Dias M. . Effects of electroacupuncture on local anaesthesia for inguinal hernia repair: a randomised placebo-controlled trial. Acupunct. Med. 28, 65–70 (2010). 20615859 10.1136/aim.2009.000570

[b103] Zhang S. P., Chiu T. T. & Chiu S. N. Long-term efficacy of electroacupuncture for chronic neck pain: a randomised controlled trial. Hong Kong Med. J. 19 Suppl 9, 36–39 (2013). 24473589

[b104] Sangdee C. . Electroacupuncture versus diclofenac in symptomatic treatment of osteoarthritis of the knee: a randomized controlled trial. BMC Complement. Altern. Med. 2, 3 (2002). 11914160 10.1186/1472-6882-2-3 PMC102323

[b105] Johansson B. B. . Acupuncture and transcutaneous nerve stimulation in stroke rehabilitation: a randomized, controlled trial. Stroke 32, 707–713 (2001). 11239191 10.1161/01.str.32.3.707

[b106] Hopwood V., Lewith G., Prescott P. & Campbell M. J. Evaluating the efficacy of acupuncture in defined aspects of stroke recovery: a randomised, placebo controlled single blind study. J. Neurol. 255, 858–866 (2008). 18465110 10.1007/s00415-008-0790-1

[b107] White A. R., Resch K. L. & Ernst E. Randomized trial of acupuncture for nicotine withdrawal symptoms. Arch. Intern. Med. 158, 2251–2255 (1998). 9818805 10.1001/archinte.158.20.2251

[b108] Dias M., Vellarde G. C., Olej B., Teofilo Salgado A. E. & de Barros Rezende I. Effects of electroacupuncture on stress-related symptoms in medical students: a randomised placebo-controlled study. Acupunct. Med. 32, 4–11 (2014). 24113152 10.1136/acupmed-2013-010408

[b109] Lin R. T. . Electroacupuncture and rosiglitazone combined therapy as a means of treating insulin resistance and type 2 diabetes mellitus: a randomized controlled trial. Evid. Based Complement. Alternat. Med. 2013, 969824 (2013). 23983807 10.1155/2013/969824 PMC3745852

[b110] Michalek-Sauberer A. . Perioperative auricular electroacupuncture has no effect on pain and analgesic consumption after third molar tooth extraction. Anesth. Analg. 104, 542–547 (2007). 17312205 10.1213/01.ane.0000253233.51490.dd

[b111] Carlsson C. P. & Sjolund B. H. Acupuncture for chronic low back pain: a randomized placebo-controlled study with long-term follow-up. Clin. J. Pain 17, 296–305 (2001). 11783809 10.1097/00002508-200112000-00003

[b112] Lund I. & Lundeberg T. Are minimal, superficial or sham acupuncture procedures acceptable as inert placebo controls? Acupunct. Med. 24, 13–15 (2006). 16618044 10.1136/aim.24.1.13

[b113] Bäcker M. . Acupuncture in migraine: investigation of autonomic effects. Clin. J. Pain 24, 106–115 (2008). 18209515 10.1097/AJP.0b013e318159f95e

[b114] Vickers A. J. Placebo controls in randomized trials of acupuncture. Eval. Health Prof. 25, 421–435 (2002). 12449085 10.1177/0163278702238055

[b115] Lundeberg T., Lund I., Näslund J. & Thomas M. The Emperors sham - wrong assumption that sham needling is sham. Acupunct. Med. 26, 239–242 (2008). 19098696 10.1136/aim.26.4.239

[b116] Takayama M. . Is Skin-Touch Sham Needle Not Placebo? A Double-Blind Crossover Study on Pain Alleviation. Evid. Based Complement. Alternat. Med. 2015, 152086 (2015). 26064153 10.1155/2015/152086 PMC4439487

[b117] Dhond R. P., Witzel T., Hämäläinen M., Kettner N. & Napadow V. Spatiotemporal mapping the neural correlates of acupuncture with MEG. J. Altern. Complement. Med. 14, 679–688 (2008). 18684075 10.1089/acm.2007.0824 PMC2556220

[b118] Moffet H. H. Sham acupuncture may be as efficacious as true acupuncture: a systematic review of clinical trials. J. Altern. Complement. Med. 15, 213–216 (2009). 19250001 10.1089/acm.2008.0356

[b119] Zhang H., Bian Z. & Lin Z. Are acupoints specific for diseases? A systematic review of the randomized controlled trials with sham acupuncture controls. Chin. Med. 5, 1 (2010). 20145733 10.1186/1749-8546-5-1 PMC2818640

[b120] Lund I., Naslund J. & Lundeberg T. Minimal acupuncture is not a valid placebo control in randomised controlled trials of acupuncture: a physiologist’s perspective. Chin. Med. 4, 1 (2009). 19183454 10.1186/1749-8546-4-1 PMC2644695

[b121] Hrobjartsson A. & Gotzsche P. C. Placebo interventions for all clinical conditions. Cochrane Database Syst. Rev. 1, CD003974 (2010). 10.1002/14651858.CD003974.pub3 PMC7156905 20091554

[b122] Vase L. . Can acupuncture treatment be double-blinded? An evaluation of double-blind acupuncture treatment of postoperative pain. PLoS One 10, e0119612 (2015). 25747157 10.1371/journal.pone.0119612 PMC4352029

[b123] Takakura N., Takayama M., Kawase A. & Yajima H. Double blinding with a new placebo needle: a validation study on participant blinding. Acupunct. Med. 29, 203–207 (2011). 21402558 10.1136/aim.2010.002857

[b124] Takakura N. & Yajima H. A double-blind placebo needle for acupuncture research. BMC Complement. Altern. Med. 7, 31 (2007). 17925042 10.1186/1472-6882-7-31 PMC2176062

[b125] Takakura N., Takayama M., Kawase A., Kaptchuk T. J. & Yajima H. Double-blind acupuncture needle: a potential tool to investigate the nature of pain and pleasure. ISRN Pain 2013 (2013). 10.1155/2013/825751 PMC3839571 24288658

[b126] Meldrum M. L. A brief history of the randomized controlled trial. From oranges and lemons to the gold standard. Hematol. Oncol. Clin. North Am. 14, 745–760 (2000). 10949771 10.1016/s0889-8588(05)70309-9

[b127] Linde K., Niemann K., Schneider A. & Meissner K. How large are the nonspecific effects of acupuncture? A meta-analysis of randomized controlled trials. BMC Med. 8, 75 (2010). 21092261 10.1186/1741-7015-8-75 PMC3001416

[b128] Kaptchuk T. J. . Components of placebo effect: randomised controlled trial in patients with irritable bowel syndrome. B. M. J. 336, 999–1003 (2008). 10.1136/bmj.39524.439618.25 PMC2364862 18390493

