# Supplementary material for: Sham Electroacupuncture Methods in Randomized Controlled Trials
Source: Sci Rep. 2017 Jan 20;7:40837. doi: 10.1038/srep40837 (PMC5247761; doi:10.1038/srep40837)
Supplement: Supplementary Information [file srep40837-s1.pdf]

# Supplementary Information

## Sham Electroacupuncture Methods in Randomized Controlled Trials

Zi-xian Chen, Yan Li, Xiao-guang Zhang, Shuang Chen, Wen-ting Yang, Xia-wei Zheng，Guo-qing Zheng

Department of Neurology, the Second Affiliated Hospital and Yuying Children's Hospital of Wenzhou Medical University, Wenzhou 325027, China.

The Supplementary Information consists of one PDF document containing Table S1.

**Table S1. Sham electro-acupuncture methods based on needle location, degree of needle insertion and electrical stimulation.**

| Methods | Sham electro-acupuncture procedures | Reference (author , year and country) |
|---|---|---|
| Sham EA type A | Control group received the same acupuncture points as selected for the real treatment. The needles with a diameter of 0.26 mm and a length of 25 mm were placed and secured by adhesive tape, without penetrating the skin. The electrodes were connected to each other in pairs and the stimulator light was on, but no electrical current was applied. The patients in both groups were told that they may or may not feel an electrical current sensation. | Ntritsou et al. 2014 USA (18) |
| | Control group received the same acupuncture points as selected for the real treatment. Blunted telescopic placebo needles were positioned at respective acupoints touching the skin but did not penetrate into the acupoints. The needle tips were inserted inside a 2 $cm^2$ foam rubber to hid the contact point between the needle and skin. The needles were connected with electrical wire but no electrical stimulation was applied. | Chu et al. 2012 Hong Kong (19) |
| | Control group received the same acupuncture points as selected for the real treatment. The half-cut needle with a diameter of 0.26 mm and a length of 40 mm was inserted through a cube-shaped piece of elastic foam to obscure the patients' vision of the insertion points. The half-cut, blunt needle was inserted into the elastic foam and caused a pricking sensation when it touched the skin without puncture. | Wang et al. 2010 Denmark (20) |
| | Control group received the same acupuncture points as selected for the real treatment. Specially-designed Streitberger sham acupuncture needles were placed on the surface of the skin, which works like a retractable magic sword: the needle appears to be penetrating the skin, the patient sees and feels a sensation of needle penetration but the needle is actually retracted up the needle shaft. The needles were connected to a de-activated electro-acupuncture device. | Zyloney et al. 2010 USA (21) |

| | |
|---|---|
| Control group received the same acupuncture points as selected for the real treatment. The acupuncture needles used for treatment were 3cm, 30 gauge solid disposable filiform stainless steel. The sham needle was secured to the skin with a plastic ring covered by a sticking plaster. 'AS Super 4' Electro-stimulator was attached to needle, in which it produced sound signals but no electrical current. A treatment session was for a total of 30 minutes. | Jubb et al. 2008 UK (22) |
| Control group received the same acupuncture points as selected for the real treatment. A specially-designed Streiberger sham acupuncture needle was placed on the surface of the skin and connected to a de-activated electro-acupuncture device without current for 25minutes. | Kong et al. 2009 USA (31) |
| Control group received the same acupoint sites as selected for the real treatment. No needles were inserted. The electric wire was placed at the surface and connected to the electro-acupuncture device without current applied. The acupuncture was maintained throughout the operation. | Yang et al. 2012 China (33) |
| Control group had acupuncture on the therapeutic points as selected in the real electro-acupuncture group. After a standard antiseptic process, blunt-tip acupuncture needles, 10 mm in length and 0.22 mm in diameter, via a sterile plastic tube stabilized by a foam block, inserted through the foam block without penetrating the skin. The needle was connected for 45 min to the programmable electrical stimulator without electricity, despite an activated output light. | Leung et al. 2011 Hong Kong (37) |

| | |
|---|---|
| Control group had acupuncture on the same points used in real electro-acupuncture group. The sham placebo acupuncture needles (DongBang AcuPrime Acupuncture Inc., South Korea) with blunt tips were put on the skin to avoid insertion. The needles were then connected to the Han's acupoint nerve stimulator (HANS-200E, Jisheng Medical Technology Co., Ltd., Nanjing, China), but with zero electricity. | Chen et al. 2013 China (40) |
| Control group had acupunnture on the therapeutic acupoints as selected for real electro-acupncture group. The sham technique consisted of a plastic guide tube which was taped on the skin to produce discernible sensation and two bent needles which was attached on the surface without penetration and covered by adhesive tapes. The needles were pressed every 5 min to evoke pressure sensation. Alligator clips were used to connect the needle with a non-functioning electrical stimulator, which was placed on a table within subjects' eyesight, showing a flashing light. No *de qi* sensation was induced. Subjects were asked to keep in supine during the whole procedure of treatment and an object was deliberately placed at waist level to block their vision of the details of sham technique. The duration of treatment was 25 min. | Zheng et al. 2010 Australia (60) |
| Control group received the same therapeutic acupoints as selected for real electro-acupunture group. The Streitberger's placebo acupuncture needles, with parameters of 0.30×30 mm and retractable tips, were put on the surface to avoid skin insertion and *deqi* sensation. A battery-operated pulse generator was switched on to deliver the audiovisual stimuli in same manner as in real electro-acupuncture group. But the lead wires attached to the needles were concealed and disconnected to the generator so that no electrical stimulation was applied during the whole sham procedure. Both real and sham group had same treatment schedule, which consists of 12 sessions of 30-min treatment over 6 weeks. | Oh et al. 2013 Australia (66) |
| Control group received the same therapeutic acupoints as selected for real electro-acupuncture group. A homemade acupuncture needle with blunt tip was pressed to the skin to produce pinprick sensation and avoid derm penetration, and was fixed by an non-transparent fixator, which was also used in real treatment group to make the intervention given out of subjects' vision. The needle was then connected to the incorrect output socket of the electrical stimulator. Thus no electricity was passed to the needle during sham procedure. Both real and sham group had 30-min treatment twice per day for consecutive 7 days after thoracotomy. | Wong et al. 2006 Hong Kong (67) |

| | |
|---|---|
| Control group received the same therapeutic acupoints mentioned in the real electro-acupuncture group. All acupoints were covered with adhesive, transparent dress before induction of anesthesia. No needle insertion was performed during sham procedure. The electrodes instead were stuck to the skin with no lines connected to the electric-acupuncture machine during the 20-min intervention. | Kvorning et al. 2003 Sweden (73) |
| Control group received the same therapeutic acupoints mentioned in the real electro-acupuncture group. All acupoints were covered with adhesive, transparent dress before induction of anesthesia. No needle insertion was performed during sham procedure. The electrodes instead were stuck to the skin with no lines connected to the electric-acupuncture machine during the 20-min intervention. | Kvorning et al. 2003 Sweden (74) |
| Control group received the same acupoints as selected for real electro-acupuncture group. No needle insertion was applied to the skin. The wires instead were pasted over the arm to link the acupoints to the electro-acupuncture system with no electrical stimulation delivered. The limb was then covered with a drape to keep blinding. The treatment started 5 minutes before the surgery. | Sahmeddini et al. 2010 Iran (76) |
| Control group had acupuncture on same therapeutic acupoints selected for real electro-acupuncture group. The Streitberger needles were used and held by surgical tape or hair pins. The needle was blunt and contractible inside the copper handle. When its tip touched the skin, a pricking sensation was felt by the subject, thereby simulating the puncturing of the skin. The needle moved inside the handle and appeared to be shortened. The same electric stimulator was then linked to the needles to deliver pseudostimulation with zero frequency and intensity. Both real and sham group received 9 sessions of thrice-weekly treatment over 3 weeks. | Yeung et al. 2009 Hong Kong (81) |

| | |
|---|---|
| Control group received the same therapeutic acupoints as selected for real electro-acupuncture group. Electrodes were attached to corresponding skin areas and fixed by securing tapes with no needle insertion. Identical electrodes and tapes were used in real treatment group as well. For sham procedure, no connection was done between the electrode and the electro-acupuncture device. Hence no electricity would be passed to the acupoints even with the device activated throughout the procedure. A vertical drape was placed at the head of the subject to prevent the anesthesia nurse from seeing the rest. All subjects were treated for 20 minutes after anesthetic induction. | Kvorning and Akeson 2010 Sweden (94) |
| Control group received the same acupoints as selected for real electro-acupuncture group. Electrodes were attached to the skin without needles and linked to the P-Stim™ device. No electrical stimulation was applied during sham procedure. The whole ear was then covered with identical ear dressings in both groups to prevent the subjects and the assessors from observing the intervention. The treatment started 15 minutes before the end of general anesthesia and lasted 72 hours. | Holzer et al. 2011 Austria (96) |
| Control group received the same acupoints as selected for real electro-acupuncture group. A non-transparent case, in which P-Stim™ and adhesive tape were included, was used to blind the subjects and investigators. The P-Stim™ device was programmed for no electrical stimulation. The tape was placed over the whole ear with no needle attached. The treatment started 30 min before the oocyte retrieval and lasted until 1 h after the end ofsurgery. | Sator-Katzenschlager et al. 2006 Austria (97) |
| Control group received the same therapeutic acupoints as selected for real electro-acupuncture group. The method of sham transcutaneous electrical nerve stimulation (TENS) was done by using disposable paediatric electrocardiogram electrodes (Hal Ind. & Com. Ltd, São Paulo, Brazil). The stimulator was then connected to the electrodes to emit a sound single without electricity passed to the skin. All subjects were treated from 30 min before surgery until the end of surgery. | Dias et al. 2010 Brazil (102) |

| | |
|---|---|
| Control group received the same therapeutic acupoints as selected for real electro-acupuncture group. The method of sham laser acupuncture was applied using a mock laser pen. The pen was inactivated throughout the intervention with a red light emitted to blind the subjects and practitioners. Each acupoint was treated for 2 minutes, with the pen at a distance of 0.5 to 1 cm from the skin. In total 9 sessions of real or sham intervention were administered over 3 weeks. | Zhang et al. 2013 Hong Kong (103) |
| Control group received the same acupoints as selected for real electro-acupuncture group. Patch electrodes, rather than acupuncture needle, were attached to the points. The same electro-acupuncture apparatus, as in real treatment group, was then connected to the electrodes to produce a sound but without electrical stimulation. Each session lasted 20 minutes. All subjects were treated 3 times per week over 4 weeks. | Sangdee et al. 2002 Thailand (104) |
| Control group received the same therapeutic acupoints as used in real electro-acupuncture group. The method of mock transcutaneous electrical nerve stimulation (TENS) was applied for 30 minutes by using a TENS machine with red flashing lights and deactivated leads. Hence no current was passed to the adhesive electrodes attached to the points. Treatment began 4–10 days after the stroke. All subjects were treated three times a week for a period of four weeks. | Hopwood et al. 2008 UK (106) |
| Control group received sham transcutaneous electrical nerve stimulation (TENS) on similar sites as used in real electro-acupuncture group. The wired electrodes from the the electrostimulation device, which was adapt by the manufacturer to emit a beeping sound without electricity conducted, were attached to the skin via microporous medical tape. Each treatment lasted 20 min. All subjects were treated once weekly over 6-8 weeks. | Dias et al. 2014 Brazil (108) |

| | | |
|---|---|---|
| | Control group received the same therapeutic acupoints as selected for real electro-acupuncture group. Electrodes were placed on the skin with no acupuncture manipulation or electrical stimulation. All subjects received one session of 30-min intervention plus the same dose of rosiglitazone. | Lin et al. 2013 Taiwan (109) |
| | Control group received the same therapeutic acupoints as used in real electro-acupuncture group. Small metal plates were attached to the skin using adhesive tapes and then connected with the P-Stim. For sham procedure, no electrical stimulation was applied. Subjects were told that they may or not feel it. The intervention was applied about 30 min before the surgery and retained for 48 h. | Michalek-Sauberer et al. 2007 Austria (110) |
| Sham EA type B | Control group received the same acupuncture points as selected for the real treatment. Specially designed Streitberger's placebo needles were used and held by surgical tape. The blunt needle was not fixed inside the copper handle. When its tip touched the skin, a pricking sensation was felt by the subject, thereby simulating the puncturing of the skin. The needle moved inside the handle and appeared to be shortened. Electric stimulator was connected to the needles using the same stimulation modality as the real treatment group. | Chung et al. 2012 Hong Kong (23) |
| | Control group received the same acupuncture points as selected for the real treatment. The acupuncture points were covered with a plastic O-ring and secured using 4 $cm^2$ squared self-adhesive tape. Specially designed sham acupuncture needles, visually indistinguishable from verum needles, but with a blunt tip and retractable shaft, were used. The needle tip stood on the surface but did not penetrate into the skin. Electro-acupuncture device which situated behind the field of vision of the participants was connected to each needle. The parameter was the same as the real treatment. | Barlas et al. 2006 UK (24) |

| | |
|---|---|
| Control group had acupuncture on same acupoints selected for real electro-acupuncture group. Streitberger needles with dull tips were used to avoid penetration during sham procedure. These needles were quickly placed on the skin and affixed with plastic O-rings. Adhesive tapes were then stuck to the surface covering the needles. Tapes in the real treatment group were done in similar manner. The electrical stimulation at frequency of 2 Hz was delivered by an acupuncture stimulation instrument. Both real and sham group received same mode of electrical stimulation. In total 9 sessions of 30-min treatment were administered over 3 week. | Zhang et al. 2012 Hong Kong (59) |
| Control group received the same therapeutic points as selected for real electro-acupuncture group, including body and cranial acupoints. The disposable needles with diameter of 0.3 mm and length of 10-30 mm were obliquely inserted into the body acupoints to a depth of 10-30 mm. Streitberger's invasive acupuncture needles with no sharp tips were quickly attached to the cranial acupoints by adhesive tapes with no skin penetration. The same modality of electrical stimulation at frequency of 2 Hz and comfortable intensity was adopted in either real or sham electro-acupuncture group. The duration of stimulation was 30 min. In total 12 sessions of intervention were administered for 4 consecutive weeks. | Man et al. 2014 Hong Kong (65) |
| Control group received the same therapeutic acupoints used in real electro-acupuncture group. No needle insertion was applied. The electro-acupuncture device was turned on to deliver electrical stimulation of 10 Hz and 10 mA. The sites of acupuncture were then covered by curtains to blind the assessor and anesthesiologist. Only one treatment was administered for 15 min. | Meissner et al. 2004 Germany (84) |
| Control group received the same therapeutic acupoints used for real electro-acupuncture group. Needle insertion was applied without skin penetration. The needle manipulation was performed out of subjects' vision, whereas the electrical stimulator, whose indicator lights blinked as well, was placed within sight of subjects. The stimulator was connected with the needles to deliver make-and-break waves with dense wave at 4 Hz and disperse wave at 20 Hz for 30 min. Acupuncture was conducted at the end of the latency period of the first labour stage in all subjects, when the dilatation of cervix was at 3 cm. | Ma et al. 2011 China (88) |

| | | |
|---|---|---|
| | Control group received two pairs of electrodes which were attached to the sites corresponding to the therapeutic acupoints selected for real electro-acupuncture group. The electro-acupuncture equipment was then linked to the electrodes to deliver electrical stimulation at a high frequency of 80 Hz and at a fixed amplitude of 0.4 mA with subliminal intensity below the perception threshold. Each treatment lasted 30 minutes. All subjects were treated twice per week for 10 weeks. | Johansson et al. 2001 Sweden (105) |
| Sham EA type C | Control group received the same acupuncture points as selected for the real treatment. A stainless steel acupuncture needle with a diameter of 0.3 mm and a length of 40 mm was inserted into the subcutaneous tissues of the scalp in the projection of motor and sensitive areas of Penfield homunculus as well as frontal and temporal associative areas. Electrical stimulation was administered, through disconnected cables applied to the scalp, using the same parameters of the real treatment. During stimulation, audiovisual feedback was evident to patients. | Hsing et al. 2012 Brazil (28) |
| | Control group had acupuncture on the acupoints same as the real electro-acupuncture group. The disposable sterile acupuncture tube needles with the length of 2—4cm, diameter of 0.2mm for head points and 0.25mm for body points were used for quick and perpendicular insertion. The needle tubes were pulled out after insertion. No manipulation was performed to avoid de qi sensation. The needles were then attached to the electric acupuncture apparatus and the current at frequency of 15 HZ and the intensity of 0 mA was applied. After 30 min of treatment the needles were pulled out and 1min of pressure on the stab site followed. The treatment was daily administered. There was a 1-day interval each weeks. The total treatment duration was 12 weeks. | Li et al. 2010 China(44) |
| | Control group had acupuncture on the points same as the real elecrtro-acupuncture group. Two stainless steel needles, serving as the positive pole and the negative pole, were inserted with a distance of about 3cm on each point. The needles was then connected to a Functions Electrical Stimulator, which was placed with the indicator light on to ensure blinding of patients. No electricity was delivered through the needles. The treatment lasted 20 min before the surgery. | Lin et al. 2002 Taiwan (47) |

| | |
|---|---|
| Control group had acupuncture on acupoints which were selected for real electro-acupuncture. An multipoint selection pen™ was used for point identification by measuring skin resistance. A P-Stim® dummy, an metal plate without battery, was connected to sterile, 0.36 mm × 3 mm single use permanent ear needles. Subjects received treatment for 4 days per week. The total duration of treatment was six weeks. | Schukro et al. 2014 Austria (49) |
| Control group received the same acupuncture point as selected for the real electro-acupuncture group. The filiform acupuncture needles, with length of 40mm and diameter of 0.38 mm, were perpendicularly inserted into the skin. The depth of penetration was 1-2 cm. No stimulation was applied during the sham electro-acupuncture procedure. The intervention was performed throughout the surgery, which lasted about 3-4 hour. | Xie et al. 2014 China (51) |
| Control group had acupuncture on same therapeutic acupoints selected for real electro-acupuncture group. The austenitic stainless steel needles with sizes of 26 to 30 gauge and length of 0.5-2.5 inches were inserted into the skin to the same depth as applied to the treatment group. No electrical stimulation was applied to the acupoints in sham group. The duration of each treatment was 30 minutes. In total 5 sessions were daily administered before the surgery. | Yang et al. 2010 China (75) |
| Control group receive the same two acupoints used in real electro-acupuncture group. After 75% ethanol sterilization, the skin penetration to the same depth was applied on a therapeutic acupoint and a nonacupoint located 3 cm beside the therapeutic acupoint by using two 30 gauge stainless steel acupuncture needles. No *deqi* sensation was elicited during the needle insertion. An electrostimulator was then connected to subjects via a negative pole linked to the therapeutic acupoint and a positive pole linked to the nonacupoint. However, when the instrument was turned on, no electricity was conducted to the needles. Only one session of 20-min intervention was performed before Extracorporeal Shock Wave Lithotripsy. | Chen et al. 2014 Taiwan (82) |

| | | |
|---|---|---|
| Sham EA type D | Control group received the same acupuncture points as selected for the real treatment. Stainless steel acupuncture needles with a length of 30 mm were inserted superficially just under the skin, where they were left for 30 minutes. No electrical or manual stimulation was applied to the needles. | Gosman-Hedström et al. 1998 Sweden (29) |
| | Control group had acupuncture on the same points as in the real electro-acupuncture group. Four Duoderm pads with thickness of 5mm and a size of 1cm×1cm were applied to the points. The 30 mm×0.25 mm single-use, sterile, stainless acupuncture needles with guide tubes were inserted into the Duoderm pads. Manual twirling was used during the insertion. The depth of penetration was 3-4mm. To ensure no electricity transmission to the skin, the care was taken during insertion to avoid touching the skin. Subjects were told that the Duoderm pads were for the purpose of point identification and that they might or not feel any needle sensation. The needles were then connected to an electro-acupuncture device, whose output light was acitive in a manner similar to real group. The procedure lasted 30 minutes. | Yu et al. 2013 Hong Kong (43) b |
| | Control group received the same acupoints as selected for real electro-acupuncture group. The method of minimal acupuncture was conducted. The depth of needle insertion was not more than 0.05-0.2 cun and manual manipulation was prohibited after penetration. No *de qi* sensation was elicited during sham electro-acupncture procedure. The needle were then connected to a device with disconnected electrical stimulator. In total 12 sessions of 20-min treatment were performed over 6 weeks. The same treatment schedule were followed in both real and sham electro-acupuncture group. | Wang et al. 2014 Taiwan (62) |
| | Control group received the same therapeutic acupoints as selected for real electro-acupuncture group. The needles were superficially penetrated into the skin and the depth of insertion was limited to 4 mm to avoid *deqi* sensation. The electro-acupuncture machine was then linked to the needles to deliver no electrical stimulation. Both real and sham electro-acupunture group received the same course, consisted of 8 sessions of 20-min treatment over 4 weeks. | Chan et al. 2014 Taiwan (64) |

| | | |
|---|---|---|
| | Control group received the same therapeutic acupoints as selected for real electro-acupuncture group. The acupuncture needles with identical sizes of 0.3×50 mm were superficially inserted into skin to the depth of 1-2 mm. After needle insertion, the Patheon Electro Stimulator 4-C, which was encased in a sealed box to blind researchers and was placed at the foot of bed to be out of subjects' vision, was linked to the needle to deliver no electricity. All subjects were told that they might or might not feel the vibration. The needles were retained for the duration of lithotripsy. | Wang et al. 2007 USA (72) |
| | Control group had acupuncture on the same therapeutic acupoints as selected for real electro-acupuncture group. Sterile disposable needles with parameters of 0.25 mm × 25 mm and 0.25 mm × 40 mm were used for all subjects. The superficial needle insertion to a depth of 2 mm was done with needle mounted through a 2 cm cube of foam material. After skin penetration the needles were immediately drawn back. Hence the needle tips were covered by the foam cubes and the level of needle entry was out of subjects' vision. The needles were then linked to the electrical current generator with no electricity conducted. Both real and sham electro-acupuncture group received intermittent non-specific manual twirling. All subjects were treated twice weekly for 40 minutes over the treatment period of 10 weeks. | Tam et al. 2007 HongKong (93) |
| Sham EA type F | Control group received acupuncture at point P2 and a neutral point, both of which were on the same meridian but outside the dermatome of P6, or C5, do not exert the same antinausea effect that P6 exibits. The disposable acupuncture needles with a diameter of 0.25 mm and a length of 30 were inserted to to a depth of 1 cm at the P2 point. A second needle was inserted at a neutral point. Needles were bent to lay flat against the skin, and the top end of the needle was taped to the skin. The needles were connected to the leads with an electro-acupuncture unit. The arm was then covered with full-length, soft restraints so the needle positions could not be seen. After placement of needles, the patient was allowed to emerge from anesthesia. Stimulation of the needles at low frequency, 4 Hz, was continued for 20 min as soon as the patient was awake. | Rusy et al. 2002 USA (38)a |

| | |
|---|---|
| Control group received acupuncture on non-specific acupoints located in same spinal segment as that selected for real electro-acupuncture group. Two stainless sterilized, disposable acupuncture needle with parameters of 40×0.25 mm were perpendicularly and bilaterally inserted into the surface to a depth of 25-40 mm. Manual manipulation, including lift, thrust and rotation, was conducted for about 1 min in both real and sham electro-acupuncture group to evoke *de qi* sensation. Then another two needles were superficially penetrated on another two non-acupuncture points which were 2 mm above mentioned acupoints. Four needles were connected to a electric stimulating device via four clips and two lines. Electrical stimulation at tolerable intensity and frequency of 2/100 Hz was daily applied for 10 min in first session and 30 min in next two sessions. | Ma et al. 2010 China (61)a |
| Control group received nonspecific acupuncture acupoint which was located near the therapeutic acupoint but uncorrelated with postpartum insufficient lactation according to Chinese medicine theory. The skin penetration, with a 10° to 15°angle between the needles and the surface and with a depth of 0.2 cun in the direction of the wrist joint, was performed by using the 0.5 cun, disposable acupuncture needles. An electrical stimulator was then linked to the needles to deliver intermittent wave with tolerable intensity and 20 Hz frequency. Both real and sham electro-acupuncture group had 2 courses of treatment over 2 weeks. Each course consisted of 5 once-a-day treatments with a two-day interval between courses. | Wei et al. 2008 China (71) |
| Control group had acupuncture at nonspecific acupoint which was neither in the same meridian nor in the same nerve segment with the therapeutic acupoint selected for real electro-acupuncture group. No literatures had reported its use for treating migraine. The vertical needle insertion was applied to the skin with a depth of 20-30 mm. Needle sensation was introduced by twirling, lifting and thrusting. An electrical stimulator was then linked to the needle ends to deliver electrical stimulation at 100 Hz frequency and at tolerable intensity for 30 min. Both groups received 4 courses of intervention. Each course consisted of 5 sessions of once-daily treatment with a 2-day interval between courses. | Jia et al. 2009 China (78) |

| | |
|---|---|
| Control group received nonspecific acupoint, Xuanzhong (GB39), which was deemed unrelated to the treatment of primary dysmenorrheal. An HanYi disposable acupuncture needle with size of 0.25 mm×40 mm was inserted into GB39. Another needle with size of 0.25 mm ×25 mm was placed about 0.5 cm far from GB39. The depth of needle insertion was same in both groups. Han's Acupoint Nerve Electro-stimulator, HANS-200, was then linked to the needles by using a pair of bipolar stimulating electrodes. The electrical stimulation was applied at alternative frequency (2/100 Hz) with current amplitude ranging from 0.5 mA to 1.6 mA. The intensity of stimulation was set midway between the sensory and pain thresholds. The duration of each session of treatment was 30 min. All treatments begin in 24 hour after menstruation and were administered once daily for consecutive 3 days. | Liu et al. 2011 China (98) a |
| Control group received nonspecific acupoint, Xuanzhong (GB39), which was deemed unrelated to the treatment of primary dysmenorrheal. An HanYi disposable acupuncture needle with size of 0.25 mm×40 mm was perpendicularly inserted into GB39. Another needle with size of 0.25 mm ×25 mm was placed about 0.5 cm far from GB39. The needle insertion to the same depth of 10-30 mm was done in both groups. Han's Acupoint Nerve Electro-stimulator, HANS-200, was then linked to the needles by using a pair of bipolar stimulating electrodes. The electrical stimulation was applied at alternative frequency (2/100 Hz) with current amplitude ranging from 0.5 mA to 1.6 mA. The intensity of stimulation was set midway between the sensory and pain thresholds. The duration of each session of treatment was 30 min. All treatments begin in 24 hour after menstruation and were administered once daily for consecutive 3 days. | Liu et al. 2014 China (99) a |
| Control group received nonspecific acupoint, GB39, which was rarely used to relieve dysmenorrheal according to the theory of Traditional Chinese Medicine. An sterile, single-use acupuncture needles with size of 0.25×40 mm was perpendicularly inserted to a depth of 0.5 to 1 cun at above point. Manual manipulation including thrusting, lifting and rotating was performed until "deqi" sensation was elicited. Another needle was placed about 0.5 cm beside the point in the proximal direction. The electro-acupuncture needle device (HANS's model, LH202H) were then linked to the two needles to deliver one session of 30-min electrical stimulation at a frequency of 2/100 HZ. | Shi et al. 2011 China (101) a |

| | | |
|---|---|---|
| Sham EA type L | Control group received non-acupoints which were 1-2cm away from the meridian therapeutic acupoints. Stainless steel acupuncture needles with a diameter of 0.25 mm and a length of 25 mm were introduced perpendicular to the skin, advanced to a depth of around 20mm but without sparrow pecking. Electric stimulator was connected to the needles. Electrical stimulation was administered at a pulse width of 200 ms and at a frequency of 1-4 Hz until the patient perceived the current, after which it was switched off. This group of patients were told on the first day of the treatment that the perception of current would disappear through habituation. | Sahin et al. 2010 Turkey (26) |
| | Control group had acupuncture bilaterally on the inactive symmetrical points near the active points. A stainless steel acupuncture needle with a diameter of 0.25 mm and a length of 25 mm was inserted into the skin. the needles were electrically stimulated at a frequency of 100 Hz, the highest intensity of stimulating current that the patient could bear without pain. | Fanti et al. 2003 Italy (27) |
| | Control group had acupuncture at the sites distant to the traditionally recognized acupoints or meridians lines. The locations were described as follows: Sham-point 1:The medial side of the arm at the anterior border of the insertion of the deltoid muscle at the junction of the deltoid and biceps muscles; Sham-point 2:The edge of the tibia ,1-2 cm lateral and horizontal to the Zusanli, ST36; Sham-point 3: On the ulnar side of the arm, half way between the epicondylus medialis of the humerus and the ulnar side of the wrist. After sterilization, the sterile disposable acupuncture needles with a diameter of 0.30 mm and a length of 40 mm were inserted transversely or obliquely into to the points to a depth of 15- 30 mm. The auxiliary needles were perpendicularly punctured 2 mm lateral to the points, to 2 mm in depth. After needle insertion, No manual manipulation was administered to avoided *De qi* sensation. The needles were then connected to the electrodes of acupoint nerve stimulator. The frequency of stimulation was 100 Hz, and the intensity of the electricity stimulus was manually adjusted to a comfortable sensation. The needles were retained in body for 30 min. | Yang et al. 2014 China (39) |

| | |
|---|---|
| Control group received acupuncture on the points at 10 mm below each patellar, into the patellar tendons where there are no acupuncture points. The 30 mm×0.25 mm single-use, sterile, stainless acupuncture needles with guide tubes were inserted to the depth of 10mm. Manual twirling was used during insertion. Subjects were told that they might or not feel any needle sensation. The needles were then connected to an electro-acupuncture device to provide current in a manner similar to the real group. The treatment lasted 30 minutes. | Yu et al. 2013 Hong Kong (43) a |
| Control group had acupuncture on the sites which were 7-12 Cun away from real acupoints. The needle insertion was performed with the depth of 0.75-1.5cm which was same as the real electro-acupucnture group. A HANS acupoint nerve stimulator was connected to the needles to supply current at frequency of 2 Hz/100 Hz. The intensity of stimulation was adjusted to the maximal tolerance of subjects. The treatment was maintained throughout the surgery. | Li et al. 2013 China (46) |
| Control group received acupuncture on the sites which were a proper distance away from the acupoints mentioned in the real electro-acupuncture group. The acupuncture needles were slightly inserted into the subjects' skin and then quickly removed. The electrical stimulator was used to supply current just at the beginning of the intervention. Once subjects perceived the electrical stimulation, the stimulator was powered off. Each session of treatment lasted 20 min. A total of eight sessions were performed over 8 weeks. | Chen et al. 2013 Taiwan (48) |
| Control group had acupuncture on two sham acupoints which were devised to avoid any known meridian or extra-point. The needles were inserted into the skin to the depth same as corresponding real acupoints. A battery-operated stimulator was used to supply strong but comfortable electrical stimulation at frequency of 18 Hz/3.85 Hz. Both real and sham electro-acupuncture group received treatment throughout the operation which lasted 3-4 h. | Yu et al. 2014 China (50) |

| | |
|---|---|
| Control group had acupuncture on the sites which were 1 cm away from the acupoints used for the real electro-acupuncture group. The 0.30 mm × 33 mm needles were placed on the sham points, which was same as the real electro-acupuncture group. The same electro-acupuncture machine were then connected to the needles to supply same oscillation waves as the real treatment group. A total of 18 sessions of 45 min of intervention were performed for 6 week. | Song et al. 2009 China (53) |
| Control group had acupuncture on non-acupoints which were away from any known ear acupoint. The sterile stainless steel acupuncture needle with a length of 2 inches were obliquely inserted into the skin after disinfection. The depth of penetration was 1-2mm. The electrical stimulation with dense-disperse waves was applied via connecting needles to a 3-channel electro-acupuncture instrument. The intensity of stimulation was adjusted to be just below the threshold for pain. In total 15 sessions of 25min of intervention were administered for consecutive 3 weeks. | Shafshak 1995 Egypt (54) |
| Control group had acupuncture on non-acupoint located at the midpoint between the Stomach and Gall-bladder Meridians in the level of SP6 and GB39, the therapeutic acupoint and non-specific acupoint selected for real and the other sham electro-aupuncture group, respectively. Two stainless sterilized, disposable acupuncture needle with parameters of 40×0.25 mm were perpendicularly and bilaterally inserted into the surface to a depth of 25-40 mm. Manual manipulation, including lift, thrust and rotation, was conducted for about 1 min in both real and sham electro-acupuncture group. But no de qi sensation was produced during the procedure. Then another two needles were superficially penetrated into another two non-acupuncture points which were 2 mm above mentioned acupoints. Four needles were connected to a electric stimulating device via four clips and two lines. Electrical stimulation at tolerable intensity and frequency of 2/100 Hz was daily applied for 10 min in first session and 30 min in next two sessions. | Ma et al. 2010 China (61)b |
| Control group received non-acupoint which was 1 cm beside the right corner of the mouth. An sterilized stainless steel acupuncture needle with length of 1.5 cun was introduced to the non-acupoint. Another 0.5 cun acupuncture needle was inserted 1 cm away from this non-acupoint. No *deqi* sensation was elicited during insertion. The same depth of skin penetration was applied in either real or sham electro-acupuncture group. The needles were then connected to the LH202 type Han's point stimulator via two shielding electric wires. The stimulator was placed outside the treatment room. Electrical | Wang et al. 2007 China (77) |

| | |
|---|---|
| stimulation was administered at a frequency of 5 Hz and at intensity of 1-3 mA with no pain perceived by subjects. | |
| Control group received non-acupoints which were 2 cun lateral to the corresponding acupoints selected for real electro-acupuncture group. The skin penetration was applied to a depth of 6-8 cm and at an angle of approximate 45°by using the needles with size of 0.30×100 mm. The GB6805-2 Electro-Acu Stimulator was then linked to the acupoints through the electrodes attached to the needle handles. Disperse-dense wave, 20 Hz electric current at maximum but tolerable intensity was used for both real and sham groups. Subjects in both groups had a total of 16 treatments, which consisted of five once-daily treatments in first and second week; three every-other-day treatments in third and forth week . | Wang et al. 2013 China (79) |
| Control group received acupuncture on a nonacupoint at the top one third and a nonacupoint at the lower one third length of muscle belly of the tibialis anterior, respectively, and 3 cm laterally to the anterior crest of the tibia, avoiding any known meridian or acupoint. The vertical needle insertion to the same depth of 20-30 mm as that for real acopoints was applied without *deqi* sensation via two 0.3×50-mm acupuncture needles. An electro-acupuncture apparatus (SDZ-II)[b] was then connected to the needles to deliver constant-current square-wave pulses at 40Hz frequency and 1ms pulse width, with the maximal intensity the participants could tolerate. The negative pole was linked to the proximal needle and the positive pole to the distal needle. In total 18 sessions of treatment, which consisted of three 15-min treatments in first week, three 20-min treatments in second week and twelve 30-min treatments in week 3 through 6, were administered over 6 weeks, | Zhou et al. 2012 China (85) |

| | |
|---|---|
| Control group received nonacupoints which were 7-12 cun above the corresponding therapeutic acupoints selected for real electro-acupuncture group. The needle insertion was applied with the depth of 0.75-1.5 cm. The needles were then linked to an LH202H HANS acupuncture point nerve stimulator to deliver electrical stimulation at alternative frequency of 2 Hz/100 Hz and at maximal intensity that subjects could tolerate. The treatment lasted from the induction of anaesthesia until the end of the surgery. | Li et al. 2013 China (89) |
| Control group received nonacupoint located on lateral side of lower leg, 3 inches above the tip of external malleolus and 1.5 inches behind anterior crest of the tibia. An HanYi disposable acupuncture needle with size of 0.25 mm×40 mm was perpendicularly inserted into above acupoint. Another needle with size of 0.25 mm ×25 mm was placed about 0.5 cm far from the point. The needle insertion to the same depth of 10-30 mm was done in both groups. Han's Acupoint Nerve Electro-stimulator, HANS-200, was then linked to the needles by using a pair of bipolar stimulating electrodes. The electrical stimulation was applied at alternative frequency (2/100 Hz) with current amplitude ranging from 0.5 mA to 1.6 mA. The intensity of stimulation was set midway between the sensory and pain thresholds. The duration of each session of treatment was 30 min. All treatments begin in 24 hour after menstruation and were administered once daily for consecutive 3 days. | Liu et al. 2011 China (98) b |
| Control group received nonacupoint located on fibular side of lower leg, at the midpoint between the stomach and the gallbladder meridians, 3 B-cun above the tip of external malleolus, anterior to GB39. An HanYi disposable acupuncture needle with size of 0.25 mm×40 mm was perpendicularly inserted into above acupoint. Another needle with size of 0.25 mm ×25 mm was placed about 0.5 cm far from the point. The needle insertion to the same depth of 10-30 mm was done in both groups. Han's Acupoint Nerve Electro-stimulator, HANS-200, was then linked to the needles by using a pair of bipolar stimulating electrodes. The electrical stimulation was applied at alternative frequency (2/100 Hz) with current amplitude ranging from 0.5 mA to 1.6 mA. The intensity of stimulation was set midway between the sensory and pain thresholds. The duration of each session of treatment was 30 min. All treatments begin in 24 hour after menstruation and were administered once daily for consecutive 3 days. | Liu et al. 2014 China (99) b |

| | | |
|---|---|---|
| | Control group received nonacupoint which was located on lateral side of lower leg, 3 cun above the tip of external malleolus and 1.5 inches behind anterior crest of the tibia. An sterile, single-use acupuncture needles with size of 0.25×40 mm was inserted to a depth of 0.5 to 1 cun at above point. Manual manipulation including thrusting, lifting and rotating was performed until “deqi” sensation was evoked. Another needle was placed about 0.5 cm beside the point in the proximal direction. The electro-acupuncture needle device (HANS’s model, LH202H) were then linked to the two needles to deliver one session of 30-min electrical stimulation at a frequency of 2/100 HZ. | Shi et al. 2011 China (101) b |
| Sham EA type M | The control group received acupuncture on the points 1cm away from the correct points used in the real electro-acupuncture group. The sterile, disposable and stainless steel needles with a length of 30 mm and a diameter of 0.25cm were inserted. The control group had the same treatment but with no connection to the alternating frequency equipment. Each session of treatment lasted 30minutes. A total of 8 sessions were performed for 4 weeks. | Aranha et al. 2015 Brazil (42) |
| | Control group had acupuncture on the non-acupoint which was 1 cm away from each therapeutic acupoint used in real electro-acupuncture group. The acupuncture needles were inserted under the skin. No electricity was passed to the needles. Both real and sham interventions were administered for 45 min every weekday morning over 6 weeks. | Song et al. 2007 USA (68) |
| | Control group received non-acupoints which were 20-30 mm far from the therapeutic acupoints selected for real electro-acupuncture group and not on meridians. The needle penetration was performed with the same depth of 1-1.5 cm. An electro-acupuncture machine was then connected to the needle. However, the electrical output of the machine was inactivated by a concealed switch before the treatment. In total 12 sessions of 30-min real or simulated electro-acupuncture were done over 6 weeks. | Cameron et al. 2011 Australia (69) |

| | | |
|---|---|---|
| Sham EA type N | Control group received predefined distant non-acupoints which were devised to avoid any therapeutic effect for insomnia in accordance with traditional Chinese medicine. The method of minimal acupuncture was used. Superficial needle insertion was performed with no *deqi* sensation elicited. The needles were then connected to a electric stimulator and the electrical stimulation at frequency of 4 Hz was delivered for 30 min. In total 9 sessions of treatment were administered over 3 weeks. | Yeung et al. 2011 Hong Kong (63) a |
| | Control group received non-acupoints which were 2 cm away from the corresponding acupoints selected for real treatment group. The shallow and horizontal needle insertion was bilaterally applied at two non-acupoints by using four single-use disposable sterile acupuncture needles. A battery-powered pulse generator was connected to the needles via microalligator clips and electrodes to deliver electrical stimulation for 30 min at 2Hz frequency and 10 mA intensity. In total 4 sessions of treatment was conducted over 2 weeks. | Wang et al. 2008 China (83) |
| | Control group received nonacupoints which were about 20 mm beside the therapeutic acupoints selected for real treatment group. The superficial needle insertion to a depth of 3-4 mm was applied with adhesive tapes. An electrostimulator (Unipuls, Seirin AG, Neu-Isenburg, Germany) was then linked to the needles to deliver electrical stimulation, which was similar to but weaker than that applied in the real treatment group. The intensity of current was adjusted to reach the threshold of perception, no more increase was done ever since. Treatment consisted of 6 session of real or sham electro-acupuncture over 3 weeks. Subjects were individually treated at different times to avoid any exchange of information. | Deluze et al. 1992 Switzerland (90) |
| | Control group received non-acupoint which was located on the medial aspect of each patella and identified by the point detector. Two 32-gauge, 1.5-inch acupuncture needles were superficially placed on bilateral non-acupoints. A portable desktop needle stimulator (AWQ-104, manufactured in China, distributed by OMS Baintree, MA, USA) was then lined to the needles to deliver intermittent, biphasic square pulse at 4 Hz frequency with perceptible and comfortable intensity. After 20 minutes of electrical stimulation, the needles were removed and a seed was placed at each non-acupoint. All subjects were instructed to press the seeds whenever they experience the desire to smoke. The duration of intervention lasted two weeks. | Waite and Clough 1998 UK (95) |

| | | |
|---|---|---|
| Sham EA type O | Control group received non-acupoint which was 2cm away from each corresponding acupoint. Stainless steel acupuncture needle with a diameter of 0.3 mm and a length of 40 mm and 25cm were inserted less than 5 mm superficially. The acupuncturist did not manipulate the needles and no de qi sensation was elicited. The needles were connected to the electro-acupuncture machine without electrical current for 20 minutes, simulating a real electro-acupuncture procedure. | Jing et al. 2009 China (30) |
| | Control group received non-acupoints which were 0.3-0.5 cun far away from the authentic points used in the treatment group. The routine needles were inserted superficially as much as possible. The needles were connected to the acupuncture machine with the disconnected electrical output lines, which looked the same as the real lines but without electricity conveyed. The needles were in place for 20 minutes. A total of 12 treatments were supplied during a treatment period of 6 weeks. | Darbandi et al. 2013 Iran (32) |
| | Control group had acupuncture bilaterally on the sites superior and lateral to the therapeutic points. The acupuncture needles diameter of 32 gauge and a length of 50mm were inserted 2 cm subcutaneously. The needles were connected to an inactive channel of the electro-acupuncture apparatus. When the stimulator was turned on, the electrical pulses were delivered to another channel not connected to the patient. Consequently, the same audiovisual stimuli was evident to the patients in the control group. The needles were left in position for 30 minutes each time. Both true and sham electro-acupuncture were performed daily for 5 consecutive days after surgery. | Zhang et al. 2014 China (35) |
| | Control group had acupuncture on non-acupoints one centimeter from the side of the points used for the real electro-acupuncture group. The disposable stainless steel needles with a diameter of 0.20 mm and a length of 30 mm were inserted less than 0.2 cm. The needles were connected to the terminals of an impulse electro-stimulator set, but without actual current given. Each session of electro-acupuncture (real or sham) lasted 30 minutes. The interventions were weekly administered for six consecutive months. | Quispe-Cabanillas et al. 2012 Brazil(41) |

| | |
|---|---|
| Control group had acupuncture on non-classical acupuncture points which were devised to avoid any known meridian or extra-point. The needle technique was gently performed with superficial penetration. A mock electro-acupuncture instrument was used to supply no electrical stimulation. Either real or sham electro-acupuncture group received two sessions of treatment each week. The treatment duration was 6 weeks. | Zheng et al. 2007 Australia (45) |
| Control group had acupuncture on non-acupoints which were 1 inch beside therapeutic acupoints but still in the same segment. The disposable, sterile stainless needles with parameter of 0.15 mm ×15 mm, which were minimal acupuncture needles in comparison with those used in the real electro-acupuncture group, were subcutaneously inserted into the skin. *De qi* sensation was avoided during penetration. The lines were attached to the needles and the electrical stimulator was manipulated with no electrical stimulation delivered. The courses of treatment were same in either real or sham electro-acupuncture group. Each treatment lasted 30 min. In total 15 sessions of intervention were applied during 7-8 weeks. | Naslund et al. 2002 Sweden (56) |
| Control group had acupuncture on non-acupoints near the therapeutically unrelated acupoints which were not far away from the acupoints selected for real electro-acupuncture group. The disposable needles with size of 36-gauge, which were same as that in treatment group were used during the sham procedure. The procedure of subcutaneous penetration was administered without manipulation or stimulation to ensure that no *de qi* sensation was evoked. The needles were then linked to a electrical stimulator, by which the same audiovisual stimuli as in real electro-acupuncture group were delivered. But no electricity was applied to the needles. Both real and sham electro-acupuncture technique were daily conducted for 5 days. Each session of intervention lasted 20 min. Before assignment, the sham electro-acupuncture technique was described as "non-classical acupuncture" to confuse subjects. They were informed that the specific benefit of either intervention was unkown. | Shen et al. 2000 US (57) |
| Control group received acupuncture on non-acupoints which were 10-15 cm laterally away from the therapeutic acupoints selected for real electro-acupncture group. Small dimension acupuncture needles with parameter of 0.25×15 mm were superficially and horizontally inserted into the skin to the depth of 0.5-1 mm. No additional stimulation was applied after needle penetration. "De qi" sensation was avoided. In total 14 sessions of 30-min treatments, which consisted of 4 sessions for first 2 weeks and | Wyon et al. 2004 Sweden (58) |

| | |
|---|---|
| 10 sessions for another 10 weeks, were administered during a period of 12 weeks. | |
| Control group received non-acupoints which were 0.3-0.5 cun far from the therapeutic acupoints selected for real electro-acupuncture group. After skin sterilization with 75 % alcohol, the acupuncture needles with length of 3.8 cm were inserted into the surface as superficially as possible. Two disconnected but indistinguishable output wires were used to connect the needles to an electrical stimulator for 20 min. Both real and sham electro-acupuncture had treatment twice per week over 6 weeks. | Darbandi et al. 2014 Iran (70) |
| Control group had acupuncture on non-acupoints which were far from any known meridian or extra-point. The superficial and oblique penetration to a minimum depth of 1 cm was applied by using 0.22 × 30-mm sterile stainless steel acupuncture needles. An control electro-stimulator, whose appearances was indistinguishable from the one used in real electro-acupuncture group, was then connected to the needles via modified leads. Thus, no current was passed to the skin when the stimulator was switched on for 30 min. Both real and sham group received 12 sessions of treatment over a period of 6 weeks. | Andreescu et al. 2011 Canada (80) |
| Control group had acupuncture on non-acupoints which were remote from any classically described meridian channel or extra-point. The sites of non-acupoints were located 3 cm laterally to the corresponding therapeutic acupoints used for real electro-acupuncture group. The oblique needle insertion to a minimum depth of about 1 cm was applied by using 30 G 3.8 cm long unused sterile acupuncture needles with a stable connection to the modified wires of an control electro-stimulator. Thus, no electrical stimulation was passed to the needles via the modified wires when the stimulator was on. All subjects received real or sham treatment for 30 min before general anesthesia. | Yeh et al. 2012 Taiwan (86) |

| | | |
|---|---|---|
| | Control group received nonacupoint which was 1 cm lateral to the corresponding therapeutic acupoint selected for real electro-acupuncture group. 30 G 2 cun long stainless steel acupuncture disposable needles were subcutaneously inserted to a depth of 2 mm with no manual manipulations or *deqi* sensation. The needles were then connected to electro-acupuncture apparatus (HC-0501, Hung-Tai Co., Taiwan) to deliver no electrical stimulation during sham procedure. In total 12 sessions of 20-min real or sham treatment were administered during the period of 6 weeks. | Yu et al. 2011 Taiwan (87) |
| | Control group had acupuncture on the sites 15 mm away from the therapeutic acupoints. The shorter needles with a diameter of 0.22 mm and a length of 13 mm were placed at a shallower depth of insertion (2 mm) to avoid de qi. The needles were connected to the incorrect output socket of the electro-acupuncture device. Patients could see the output light flashing but no current was transmitted throughout the procedure. Patients were told that the stimulation frequency selected was not perceivable by human beings. Each session of sham electro-acupuncture lasted 20 minutes. The interventions were daily performed from postoperative day 1-4. | Ng et al. 2013 Hong Kong (91) |
| | Control group received non-acupoints which were 15 mm lateral to each corresponding therapeutic acupoint used in real electro-acupuncture group. The superficial skin penetration was applied by using two types of disposable stainless steel needles, whose sizes were 40×0.25 mm and 70×0.30 mm, respectively. A pulse generator was then connected to the needles. When the generator was switched on, the sound was made to blind the subjects but with no current passed to the points. Subjects in both group were treated twice per week for 20 minutes during the period of 6 weeks. Those in real or sham electro-acupuncture group were given treatment on alternative days to avoid any crosstalk. | Lee and Lee 2009 Republic of Korea (92) |
| Sham EA type Q | Control group received non-acupoints which were 1-2cm away from the therapeutic acupoints. A sham acupuncture needle was used, and it works like a magician's sword: the patient sees and feels the acupuncture needle, but as it is applied to the skin, the needle retracts and slides up the needle shaft rather than penetrating the skin. For body points, a 1 cm-diameter plastic ring, covered and held in place with paper surgical tape, supported needles in a vertical position. Electro-acupuncture device was administered to needles, using wires that were severed and retaped so as to leave a gap, and thus not conduct electricity. | Wayne et al. 2005 US (25) |

| | |
|---|---|
| Control group received non-acupoints which were 1 cm lateral to the therapeutic points. The needles were attached to 1 cm diameter rings and then attached to the skin using surgical tape with no penetration. The electrical wire used for sham electro-acupuncture was broken, so it could not conduct electricity. The needles were left in position for 20 minutes each time. Sham electro-acupuncture was performed daily for 10 consecutive days. | Liu et al. 2013 China (34) |
| Control group had acupuncture at nonacupuncture, nontrigger points at least 5 cm from the joint where pain was perceived to be maximal. The Streitberger non-penetrating needles were used and minimally manipulated apart from their initial contact with the skin to avoided eliciting the “De Qi” sensations. Two pairs of electrodes were connected to the needles with transcutaneous electrical nerve stimulation (TENS) unit. The dial of the TENS unit was turned on to a different channel with the light blinking but with no electricity . The frequency and duration of treatments between EA and SA groups were identical. The needles were left in place for 30 minutes. A total of 10 sham treatments were administered over 8 weeks. The frequency and duration of treatments were identical between EA and SA groups. | Mao et al. 2014 USA (36) |
| Control group received non-acupoints on the insides of the arm covers. No needles were inserted. Insulated wires instead were attached to the skin and connected with the stimulator box. The stimulator was activated so its indicator light was on to maintain blinding. The arm was then covered with full-length, soft restraints to not be seen. After placement of wires, the patient was allowed to emerge from anesthesia. The wires were in place 20 min as soon as the patient was awake. | Rusy et al. 2002 USA (38) b |
| Control group had acupuncture on the bony area next to the acupoints selected for the real electro-acupuncture group. Subjects were shown a needle with a plastic tube. The tube was taped to the skin to produce some discernible sensation. The needle was taped to the point with opaque adhesive tape. The needles were then connected to a mock electrical stimulator whose lights were on but with no current applied. Subjects were told that that they may or may not feel electricity due to its very high frequency. Subjects were asked to keep supine and close their eyes. Either real or sham electro-acupuncture treatment was preoperatively conducted and lasted 45 min. | Sim et al. 2002 Singapore (52) |

| | |
|---|---|
| Control group had acupuncture on non-acupoints on all four extremities to avoid any known acupoints or merdians. A validated Park sham device, which consisted of a dull needle and a park tube, was attached to the skin with double-sided, donut-shaped adhesive tape. The dull needle was applied to ensure no skin penetration. And the tube was devised to hide the sham insertion from subjects. Alligator clips were then attached to the needles and a electrical machine was switched on to supply no active current. | Franasiak et al. 2012 USA (55) |
| Control group received non-acupoint which was 1 inch away from each therapeutic point selected for real electro-acupuncture group. Placebo needles with blunt tips and guide tubes were used. When their tips touched the surface, the needles moved into the tubes and looked being shortened. Thus skin penetration was avoided and a pricking sensation was produced, which made sham electro-acupuncture more credible in terms of perception. The needles were then connected to a electric stimulator which delivered electrical stimulation at zero frequency and intensity for 30 min. In total 9 sessions of treatment were administered over 3 weeks. | Yeung et al. 2011 Hong Kong (63) b |
| Control group received a pair of non-acupoints, which were located 2 cun and 1 cun distal and slightly volar to acupoint SI-7, respectively. MRI-compatible blunt-tipped titanium needles were attached to above non-acupoints using a single tap without skin penetration. The needles were then connected to a constant current electro-acupuncture device (HANS LH202H, Neuroscience Research Center, Peking University, Beijing, China) via electrodes. But no electrical stimulation was applied for control group. Subjects were instructed that the intensity of stimulation was predetermined and that they may or may not feel any sensation during the procedure. All subjects were asked to keep their eyes closed and focus on the stimulation while remaining as still as possible. | Maeda et al. 2013 USA (100) 1,2 |
| Control group received non-acupoint located on the center of the mastoid bone. Either similar needles as in real electro-acupuncture group or transcutaneous electrical nerve stimulation (TENS) pads were placed superficially on the points and fixed with adhesive tapes. An inactivated electro-acupuncture instrument with flashing indicator lights was then connected to either needles or pads by using leads without current passed through. The duration of each treatment was 20 min. All subjects were treated on day 1, 3, 7. | White et al. 1998 England (107) |

| | |
|---|---|
| Control group received two large electrodes attached to the most painful area, where no acupuncture point exits. A mock transcutaneous electrical nerve stimulation (TENS) was done by using a disconnected stimulator, which was a gradient-recalled acquisition in a steady state with flashing lamp visible to subjects. Thus no electricity was passed to the points. All subjects were treated once a week over 8 weeks. Each treatment lasted 20 min. Two further treatments were administered at 4 month and 6 month, respectively. | Carlsson and Sjolund 2001 Sweden (111) |

Note: Sham electro-acpuncture (EA) type A: sham EA on therapeutic acupoints plus no penetration plus no electrical stimulation; Sham EA type B: sham EA on therapeutic acupoints plus no penetration plus electrical stimulation; Sham EA type C: sham EA on therapeutic acupoints plus the same depth plus no electrical stimulation; Sham EA type D: sham EA on therapeutic acupoints plus superficial penetration plus no electrical stimulation; Sham EA type F: sham EA on nonspecific acupuncture points plus the same depth plus electrical stimulation; Sham EA type L: sham EA on non-acupuncture points plus the same depth plus electrical stimulation; Sham EA type M: sham EA on non-acupuncture points plus the same depth plus no electrical stimulation; Sham EA type N: sham EA on non-acupuncture points plus superficial penetration plus electrical stimulation; Sham EA type O: sham EA on non-acupuncture points plus superficial penetration plus no electrical stimulation; Sham EA type Q: sham EA on non-acupuncture points plus no penetration plus no electrical stimulation.
